# DeePaN: deep patient graph convolutional network integrating clinico-genomic evidence to stratify lung cancers for immunotherapy

**DOI:** 10.1038/s41746-021-00381-z

**Published:** 2021-02-02

**Authors:** Chao Fang, Dong Xu, Jing Su, Jonathan R Dry, Bolan Linghu

**Affiliations:** 1 Translational Medicine, Research and Early Development, Oncology R&D, AstraZeneca, Boston, MA USA; 2 Department of Electrical Engineering and Computer Science, University of Missouri, Columbia, MO USA; 3 Christopher S Bond Life Sciences Center, University of Missouri, Columbia, MO USA; 4 Department of Biostatistics, Indiana University School of Medicine, Indianapolis, IN USA; 5 Wake Forest Baptist Comprehensive Cancer Center, Winston-Salem, NC USA

**Keywords:** Machine learning, Non-small-cell lung cancer, Cancer genomics, Immunotherapy

## Abstract

Immuno-oncology (IO) therapies have transformed the therapeutic landscape of non-small cell lung cancer (NSCLC). However, patient responses to IO are variable and influenced by a heterogeneous combination of health, immune, and tumor factors. There is a pressing need to discover the distinct NSCLC subgroups that influence response. We have developed a deep patient graph convolutional network, we call “DeePaN”, to discover NSCLC complexity across data modalities impacting IO benefit. DeePaN employs high-dimensional data derived from both real-world evidence (RWE)-based electronic health records (EHRs) and genomics across 1937 IO-treated NSCLC patients. DeePaN demonstrated effectiveness to stratify patients into subgroups with significantly different (*P*-value of 2.2 × 10^−11^) overall median survival of 20.35 months and 9.42 months post-IO therapy. Significant differences in IO outcome were not seen from multiple non-graph-based unsupervised methods. Furthermore, we demonstrate that patient stratification from DeePaN has the potential to augment the emerging IO biomarker of tumor mutation burden (TMB). Characterization of the subgroups discovered by DeePaN indicates potential to inform IO therapeutic insight, including the enrichment of mutated KRAS and high blood monocyte count in the IO beneficial and IO non-beneficial subgroups, respectively. Our work has proven the concept that graph-based AI is feasible and can effectively integrate high-dimensional genomic and EHR data to meaningfully stratify cancer patients on distinct clinical outcomes, with potential to inform precision oncology.

## Introduction

Recently immuno-oncology (IO) therapies including checkpoint inhibitors have transformed the therapeutic landscape of non-small cell lung cancer (NSCLC)^1–3^. However, responses to IO in NSCLC are highly variable. Recent findings suggest a heterogeneous collection of genomic alterations and clinical phenotypes can influence IO response^4–6^. Thus, there is a pressing need to discover and characterize NSCLC subgroups across both clinical and genomic landscapes to advance precision IO.

Real-world-evidence (RWE)-based clinical phenotype data such as electronic health records (EHRs), which include patient exposures, lab data, diagnosis, medications, and clinical outcomes, represent a promising resource for precision oncology. EHR-derived data have been used to identify patient subgroups to inform cancer therapeutics^7–12^. Distinct molecular subtypes^13–18^ derived from rich genomic resources, including high tumor mutational burden (TMB) and high PDL1 protein expression, have also been associated with beneficial responses to checkpoint inhibitor therapies in NSCLC^1,19–21^. The integration of both genomic and EHR evidence is expected to reveal a fuller description of tumor and patient characteristics impacting drug response. Whilst there have been many comparative studies between these high dimensional data modalities^22–24^, few studies to date integrate both genomics and EHRs for patient stratification due to all types of challenges. For instance, the study cohort can be too small to investigate this heterogeneous disease; the datasets used in subtyping studies may not be comprehensive enough to incorporate both genomic data and diverse clinical-phenotype data with long-term follow-ups; and the subtyping algorithms and models may not be effective enough to integrate high-dimensional data from both genomic and clinical domains.

Recently, artificial intelligence (AI) and deep learning methods have demonstrated great potential for discovery of cancer subtypes^25–28^, stemming from effective high-dimensional data integration and capture of complex nonlinear relationships^29–31^. However, most AI studies use a grid-based model^28,32,33^ for patient-data representation which overlook patient–patient relationships and are sub-optimal for inclusion of multiple data modalities. Graph-based patient similarity networks (PSNs) have shown promise for patient subtyping^34,35^. PSNs effectively model patient–patient relationships to intuitively enable heterogeneous data integration and to cluster patients into subtypes based on their feature similarities. Addition of deep convolutional neural networks (CNNs)-based learning of patient-data embeddings to the PSN framework holds great potential to augment patient subtype discovery through integrative usage of both genomic and EHR data.

Graph convolutional networks (GCNs)^36^ are such an efficient variant of CNNs operated on a network (i.e. graph) like PSN’s. GCNs offer fast and scalable classification of nodes in a graph through graph embedding and convolutional operations. GCN has demonstrated promise in multiple biomedical applications such as protein interface prediction and side effects prediction^37^. We sought to explore the feasibility and effectiveness of applying GCN for patient subtype discovery through integrative usage of EHR and genomic data.

We developed a data-driven, unsupervised, graph-based AI representation we call “DeePaN” (i.e. deep patient graph convolutional network) to stratify NSCLC patients, integrating 100 EHR and genomic data features from the Flatiron Health and Foundation Medicine NSCLC “clinico-genomic” database^38^ across a cohort of 1937 IO-treated NSCLC patients. Our “DeePaN” framework employs a GCN autoencoder (AE) to learn a patient-similarity-graph-based feature representation, followed by graph spectral clustering for patient subgrouping.

The “DeePaN” framework stratified patients into subgroups with distinct outcomes post-IO therapy, and this stratification was most significant when both genomic and EHR data modalities were integrated. Median survival was 9.42 months from sub-groups with poor survival vs. 20.35 months for the subgroup with better survival (*P*-value of 2.2 × 10^−11^). Comparatively, patient sub-groupings derived through well-established methods such as AE, uniform manifold approximation and projection (UMAP), and t-distributed Stochastic Neighbor Embedding (t-SNE) showed no significant difference on IO therapy outcome. Furthermore, we demonstrated the potential to use this DeePaN grouping to augment the clinical utility of an emerging IO biomarker, TMB. Characterization of the subgroups discovered by DeePaN indicates potential to inform IO therapeutic insight, including the enrichment of KRAS mutations and high blood monocyte count in the IO beneficial subgroup and IO non-beneficial subgroup, respectively.

“DeePaN” represents a graph-based AI framework with advances of effectively integrating heterogeneous clinico-genomic data modalities, leveraging graph embedding to intuitively model patient–patient relationships, and incorporating the high-performance of AI to capture complex relationships of patient data. Our work demonstrates the feasibility and effectiveness of employing a graph-based AI approach to integrate RWE-based high-dimensional EHRs and genomics to stratify NSCLC patients by IO benefit. The subtypes discovered in this work may cast new light on understanding the heterogeneity of IO treatment responses, and pave ways to inform clinical decision making and therapeutics insight for precision oncology.

## Results

### Building an IO-treated NSCLC cohort with linked clinico-genomic data

**Fig. 1:**
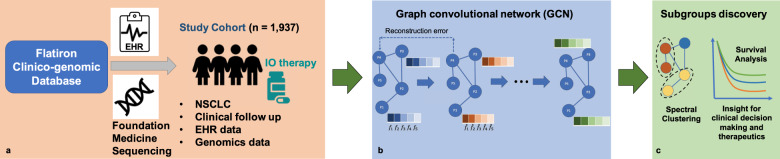
The conceptual “DeePaN” framework as a deep patient graph convolutional network integrating electronic health records and genomics to strategy NSCLC patients benefiting from immunotherapy. **a** An IO-treated NSCLC cohort (*N* = 1937) was identified from Flatiron clinico-genomic database with linked EHRs and genomics data. The clinical and genomic features are preprocessed (see “Methods” section for details) and concatenated as raw patient-data representations. **b** The raw patient-data representations are modeled by a deep patient graph convolutional network (GCN) implemented as the marginalized graph autoencoder (MGAE) to learn latent patient representations. In GCN modeling, patients are represented as nodes, and patients with similar clinico-genomic features are linked by edges. Multiple layers of graph convolutional network are stacked to learn latent patient representations, with each layer of the graph neural network being trained to produce a high-level patient-data representation from the output of the previous layer. **c** The graph-based deep patient representations are then subjective to spectral clustering to discover patient subgroups with distinct immunotherapy outcomes to inform precision-oncology including patient stratification by IO benefit.

The aim of this study is to explore the feasibility and effectiveness to develop a data-driven, unsupervised, graph AI-based “deep patient graph” (DeePaN) framework integrating genomics and EHRs to stratify NSCLC patients into subgroups useful for precision immunotherapy. Using Flatiron NSCLC clinico-genomic database, we identified an IO-treated cohort of 1937 patients characterized by 100 clinico-genomic features to develop and test this framework (“Methods” and Fig. 1). The cohort’s overall clinical and demographic characteristics are shown in Table 1 and tumor genomic characteristics are shown in Supplementary Fig. 1C.

**Table 1:** Baseline demographic and pathologic characteristics.

Characteristics	Values
Number of patients	1937
Age (year)	
Median, MAD	69.0, 10.4
Range	26.0–85.0
Sex: number (%)	
Male	984 (50.8)
Female	953 (49.2)
Race: number (%)	
African American	144 (7.4)
White	1428 (73.7)
Asian	46 (2.4)
Other Race	143 (7.4)
Histology: number (%)	
Non-squamous cell carcinoma	1443 (74.5)
Squamous cell carcinoma	419 (21.6)
NSCLC histology NOS	75 (3.8)
Stage: number (%)	
Stage I	164 (8.5)
Stage II	122 (6.3)
Stage III	372 (19.2)
Stage IV	1241 (64.1)
ECOG score: number (%)	
0	375 (19.4)
1	856 (44.2)
2	273 (14.1)
3	50 (2.6)
4	2 (0.1)
Smoking status: number (%)	
History of smoking	1657 (85.5)
No history of smoking	276 (14.2)
Previous treatment: number (%)	
No	718 (37.1)
Yes	1219 (62.9)

*MAD* median absolute deviation, *ECOG* Eastern Cooperative Oncology Group.

### Overview of the conceptual DeePaN framework

Figure 1 illustrates the overall conceptual DeePaN framework. DeePaN employs a graph representation to summarize patient data in an unsupervised AE, hereon referred to as the graph autoencoder (GAE). Specifically, each node in the graph represents a patient with node contents composed of “clinico-genomic” (combined genomic and EHR-derived clinical) features; linked neighbor patient nodes share similar clinico-genomic features. The GAE employs a “denoising process” to learn a graph embedding by allowing node content to interact with network features (“Methods”). The addition of denoising with the GAE is referred to as the marginalized graph autoencoder (MGAE)^39^. After application of MGAE-based graph embedding, a graph-based spectral clustering was then applied to discover patient subgroups with differential IO-treatment benefit.

### Patient subgroups from DeePaN show distinct IO treatment benefit

**Fig. 2:**
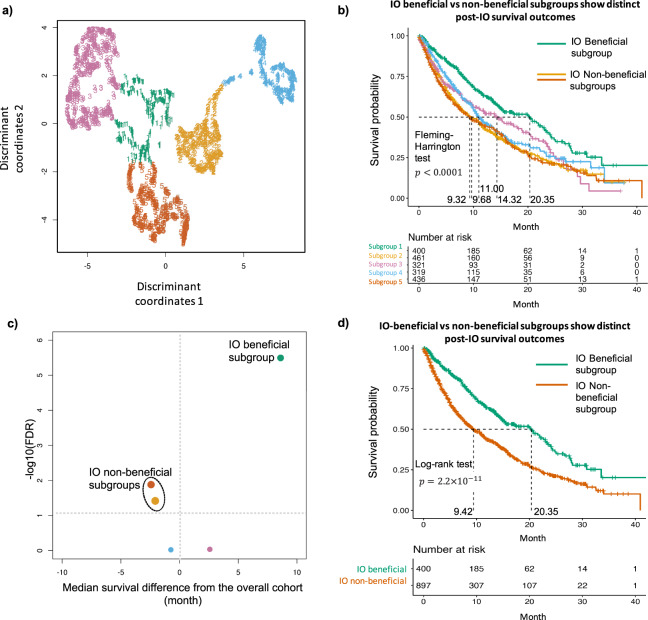
Clinico-genomic “DeePaN” framework discovered NSCLC subgroups with distinct overall survival outcomes of post-IO treatment. **a** Five distinct patient subgroups were discovered by DeePaN, visualized by the 2D UMAP projection of the deep patient graph representation in the latent space. Each data point denotes a patient and colors denote distinct subgroup memberships. **b** The five subgroups discovered by DeePaN showed overall significant post-IO survival difference by the Kaplan–Meier survival plots (same subgroup color encoding as in **a**). **c** Using the overall cohort (1937 patients) as the control, comparison of survival of each subgroup with the overall cohort identified distinct IO beneficial and IO non-beneficial subgroups, demonstrated by a volcano plot (see “Methods”). Each bubble represents a patient subgroup, same subgroup color encoding was used as in **a** and **b**, and bubble sizes are proportional to corresponding subgroup patient counts. The *x* axis represents the difference of the estimated median survival times between a subgroup and the overall cohort. The vertical line marked zero median survival difference, with bubbles on the right of the vertical line showing the tendency of beneficial IO outcomes and bubbles on the left showing the tendency of IO non-beneficial outcomes. *y* axis is the −(FDR) of the corresponding log-rank test between a subgroup vs. the overall cohort with multiple-comparison adjustment by Benjamini–Hochberg procedure, representing the statistical significance of the observed survival difference. The horizontal dashed line marked the statistical significance cutoff of FDR of 0.05. Two IO non-beneficial subgroups (red and orange) and one IO beneficial subgroup (green) were identified with significantly different post-IO overall survival from the overall cohort. We combined the two IO non-beneficial subgroups (red and orange) into one subgroup since they have similar post-IO survival outcomes. **d** The IO beneficial and the combined IO non-beneficial subgroup showed significant (*P*-value of 2.2 × 10^−11^) post-IO survival difference with estimated median survival of 20.35 months and 9.42 months, respectively, by the Kaplan–Meier survival plots.

Five distinct patient subgroups were identified (Fig. 2a) by DeePaN. Overall survival (OS) post IO treatments were compared across patient subgroups. The five subgroups showed significant OS differences (Fleming-Harrington test *P*-value <0.0001, median survival ranging from 9.32 to 20.35 months, Fig. 2b). This demonstrated DeePaN can effectively discover subgroups with distinct immunotherapy outcomes. Using the overall cohort (1937 patients) as the control, comparison of survival of each subgroup with the overall cohort identified two subgroups with poor survival, and one subgroup with better survival (Fig. 2c). Since the two poor-survival subgroups have similar post-IO OS outcomes (Fig. 2b, c), we combined them as one single IO non-beneficial subgroup (*n* = 897, 46.3% of the cohort), for comparison to the better survival group as the IO beneficial subgroup (*n* = 400, 20.7% of the cohort). We found significantly different survival post IO between the two groups (log-rank *P*-value of 2.2 × 10^−11^, median survival of 9.42 vs. 20.35 months, Fig. 2d and Supplementary Note 10). The demographic and pathologic characteristics of the IO beneficial and non-beneficial subgroups were shown in Supplementary Table 1.

### Graphical integration of EHR and genomics data is essential

**Fig. 3:**
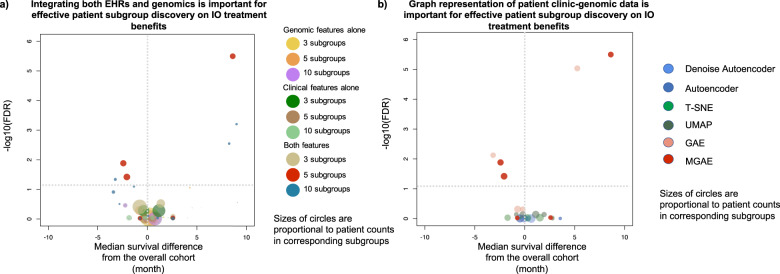
Graph representation of patient data and integration of both EHR and genomics data are essential toward identifying patient subgroups with differential IO-treatment benefits. In both volcano bubble plots, each bubble represents a patient subgroup, the *x* axis represents the difference of the estimated median survival times between a patient subgroup and the overall cohort as control. The vertical line marked zero median survival difference, with bubbles on the right of the vertical line showing the tendency of beneficial IO outcomes and bubbles on the left showing the tendency of IO non-beneficial outcomes. *y* axis is the −log10(FDR) of the corresponding log-rank test between a subgroup vs. the overall cohort with multiple-comparison adjustment by Benjamini–Hochberg procedure, representing the statistical significance of the observed survival difference. The horizontal dashed line marked the statistical significance cutoff of FDR of 0.05. **a** Integrating both EHRs and genomics is important for effective patient subgroup discovery on IO treatment benefits and setting the number of clusters (subgroups) to five outperforms cluster number of three or ten. We compared patient subgrouping using both types of features versus using EHR or genomics features alone. To make a robust comparison, we explored different number of resulting subgroups, including three, five, and ten subgroups respectively. Integrating both types of features discovers patient subgroups with significant IO beneficial and non-beneficial outcomes, while individual features alone do not identify any subgroup with significant IO beneficial or non-beneficial outcomes. In the setting of incorporating both genomic and clinical features, we obtained optimized results when the targeted number of clusters was set to 5, which is able to identify more patients with significant IO beneficial and non-beneficial outcomes and with stronger statistical confidence. **b** Graph representation of patient clinico-genomic data is important for effective patient subgroup discovery on IO treatment benefits. Subgrouping results compared with other methods demonstrates that graph representation of patient data (MGAE and GAE) discovers patient subgroups with significant IO beneficial and IO non-beneficial outcomes, while non-graph-based approaches (t-SNE, UMAP, autoencoder, and denoising autoencoder) did not identify any subgroups with significant IO beneficial or IO non-beneficial outcomes.

To evaluate whether integration of both EHR and genomics features is essential for effective identification of patient subgroups with differential IO-treatment benefits, we compared patient grouping using both types of features versus using EHR or genomics features alone (see “Methods”, “Performance evaluation on patient subgrouping by IO outcomes”). To make a robust comparison, we explored patient grouping with different numbers of resulting subgroups, including three, five, and ten subgroups, respectively. The results demonstrated that integration of both resources was essential to identify patient subgroups (Fig. 3a). This highlighted that integration of genomics and real-world clinical phenotype evidence can represent and reveal more of the determinants of cancer patient survival than using genomics or phenotype data alone.

Additionally, to investigate how (1) the patient–patient relationship-based graph topology and (2) denoising process contribute to the effectiveness to stratify patients into subgroups with differential immunotherapy outcomes, we compared four frameworks, our current MGAE which employed both the patient–patient graph topology and the denoising process, (2) the GAE which employed only the patient–patient graph topology but not the denoising process, (3) the denoising AE which employed only denoising process, and (4) the AE which employed neither (see Supplementary Note 7 for design details of these methods). The results indicate that the graph representation of patient–patient relationship is essential since only the MGAE and the GAE are capable to identify sub-groups with differential IO treatment benefits (Fig. 3b).

Many unsupervised techniques now exist that can accept multi-modal data as input. To further assess the performance of the DeePaN framework, we compared it with the commonly used tSNE^40^ and UMAP^41^ methods. The results showed that only the DeePaN framework identifies subgroups with differential survival post IO therapy (Fig. 3b). We also compared the DeePaN framework with k-medoids clustering (see Supplementary Note 6). The results showed DeePaN has better performance than k-medoids clustering by identifying more patients with significant IO beneficial and IO non-beneficial outcomes and with stronger statistical confidence (Supplementary Fig. 4). Additionally, we tested the robustness of the DeePaN framework using a ten-round “adjusted Rand index” test^42^, the result shows the framework is generally robust with a mean adjusted Rand index of 0.93 (see Supplementary Note 5).

### DeePaN discovered IO-beneficial patients with non-high TMB

**Fig. 4:**
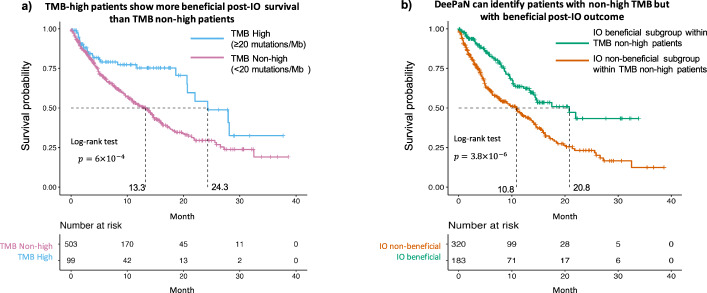
DeePaN can identify patients with non-high TMB but with beneficial post-IO outcomes. **a** High tumor mutation burden (TMB) is associated with beneficial post-IO outcomes, as observed in the overall IO cohort (log-rank *P*-value of 6 × 10^−4^, median survival of 13.3 vs. 24.3 months for TMB non-high vs. high groups, respectively). **b** DeePaN can identify patients with non-high TMB but with beneficial post-IO outcomes. Subgroups discovered by “DeePaN” are able to strategy TMB-non-high patients into subgroups with significantly differentiated survival post IO therapy (*P*-value of 3.8 × 10^−6^ from log-rank test, median survival of 20.8 months and 10.8 months, respectively), with about 10 months’ median survival difference between the IO-beneficial vs. non-beneficial group.

High TMB is an emerging biomarker utilized to enrich for patients likely to benefit from IO therapy^43,44^, as observed in our Flatiron IO cohort (log-rank *P*-value of 6 × 10^4^, median survival of 13.3 vs. 24.3 months for TMB non-high vs. TMB high groups, respectively, Fig. 4a). Many TMB non-high patients, however, may still benefit from IO therapy. We found that subtypes discovered by “DeePaN” were able to further strategy TMB non-high patients into subgroups with significantly differential survival post-IO therapy (Fig. 4b, *P*-value of 3.8 × 10^−6^ from log-rank test, median survival of 20.8 months and 10.8 months, respectively), with about 10 months’ median survival difference between the IO-beneficial vs non-beneficial group. To assess if the better post-IO survival group (green curve) in Fig. 4b has clinical-relevant beneficial IO outcomes, we used three recently FDA-approved NSCLC IO trials in 2019 and 2020 for references^45–47^. The better survival group has the median survival of 20.8 months, which is comparable with the median survivals in these recent FDA-approved IO NSCLC trials and therefore demonstrated clinical-relevant IO beneficial outcomes (see Supplementary Note 4 for details). This shows that DeePaN can identify patients with non-high TMB but with clinical-relevant beneficial post-IO outcomes with a median survival of over 20 months.

### DeePaN subgrouping shows potential to inform therapeutic insight

To inform biological insight of patient stratification with DeePaN, we characterized the IO beneficial vs. non-beneficial subgroups identified by DeePaN and identified 21 significantly enriched clinico-genomic features (Supplementary Table 2). Many features have literature evidence indicating relevance to NSCLC prognosis (Supplementary Table 3). To explore the potential of DeePaN to real new and complementary insight in comparison with classical approaches, we further explored the differences in biological insight revealed by DeePaN compared to the classical log-rank test (Supplementary Table 3). The log-rank test identified 14 significant features associated with IO outcomes with eight features in common with DeePaN. Thirteen out of 21 features enriched between DeePaN-defined subgroups did not show a statistically significant relationship to post-IO survival by log-rank, indicating the potential of DeePaN to inform insight on IO stratification complementary to the classical approach. For instance, among these 13 features uniquely enriched by DeePaN, features relevant to peripheral immune status such as high blood monocyte count and low blood lymphocyte count are associated with poor post-IO prognosis in NSCLC with supporting literatures^48–50^; KRAS mutations are enriched with the IO-beneficial subgroup^51^. There are recent literatures indicating PD-1/PD-L1 blockade monotherapy may be the optimal therapeutic schedule in NSCLC patients harboring KRAS mutations, with KRAS mutations correlating with an inflammatory tumor microenvironment and tumor immunogenicity and thus resulting in superior patient response to PD-1/PD-L1 inhibitors^51,52^. Taken together, these enriched clinico-genomic features derived from DeePaN-discovered subtypes may have potential to inform therapeutic insight on IO outcome stratification in NSCLC.

## Discussion

In this study, we explored the feasibility and effectiveness of a graph AI-based unsupervised framework, “deep patient graph” (DeePaN), to stratify IO-treated NSCLC patients from integrating rich genomics and EHR-derived clinical data. Our work has proven the concept that graphical-data-representation-based AI can effectively integrate high-dimensional genomic and EHR data to stratify cancer patients relevant to distinct clinical outcomes. This establishes the opportunity to use graph AI modeling for precision oncology.

Genomic and EHR data are two major domains of RWE generated in clinical care. Integrative modeling of these data remains challenging but holds great promise to inform precision oncology. Our work demonstrated a graph AI framework can effectively achieve clinico-genomic data integration to inform patient stratification with relevance to outcomes post-IO therapy, and is superior to data type alone and other stratification methods (Fig. 3). For instance, enrichment analysis on patient subgroups identified by DeePaN indicates that both clinical features such as blood monocyte count, blood lymphocyte count, and genomic features such as mutated KRAS are potentially associated with differential IO-treatment benefits (Supplementary Table 2 and Supplementary Note 8).

Importantly, the results demonstrate that graph representations of EHR and genomic patient data are important to discover patient sub-groups with differential IO-treatment benefits (Fig. 3b). The rationales and advantages of graph-based GCN modeling can be explained as below. First, one of the major challenges of RWE-based data analysis is the presence of noise and missingness of the data. GCN’s key concept, “neighbor aggregation”, can effectively address this challenge. In particular, the GCN method is to augment a node’s features from neighbor aggregation in a bottom-up fashion, i.e. augmenting a given patient’s clinico-genomic features from borrowing information from its similar neighbors, as a result, the clinico-genomic features of a given patient would be augmented with increased precision and less missingness. Second, GCN modeling of patient relationships also enables us to take into account the specificity of network context associated with each different neighborhood to augment a patient’s clinico-genomic features accordingly. Compared with alternative methods such as AE, which assumes all the patients are independent, GCN is expected to utilize the patient-to-patient similarity relationships more effectively and hence achieve better signal-to-noise ratio for patient subtype clustering and discovery. Third, another advantage of using GCN method stems from the fact that GCN enables utilization of the global patient network topology for effective patient subtype clustering, which takes into account all the patients and their similarities in a hierarchical structure. Through iterative graph convolution and stacking multiple layers of GCNs, the GCN method effectively enables leveraging the global network topology by integrating both the direct first neighbor and the non-direct neighbors such as the second and third neighbors with suitable weights, reflecting their relative importance at different levels of neighborhood. This is not achieved through typical graph approaches, most of which only consider conditional dependence by edges of directly neighboring nodes. Last, similar usage of topological information derived from node features for graph modeling has been proved successful in recent biomedical informatics applications such as single-cell RNA-seq data analysis^53–57^, particularly in the GCN setting^58,59^, where the edges of the cell graph were derived from kNN of gene expression profiles.

Characterization of the IO beneficial vs. non-beneficial subgroups identified by DeePaN indicates potential to inform new and complementary therapeutic insight for IO stratification in NSCLC in comparison with classical approaches such as the log-rank test approach (Supplementary Table 3). Mechanistic insight on IO outcomes in NSCLC was indicated by features significantly enriched by DeePaN-discovered patient subgroups but not reaching statistical significance by log-rank test. For instance, the enrichment of high blood monocyte count and low lymphocyte count in the IO non-beneficial group identified by DeePaN indicates that host peripheral immune status may contribute to IO outcomes; the enrichment of mutated KRAS in the IO beneficial subgroup was supported by literature evidence that KRAS mutations correlating with an inflammatory tumor microenvironment and tumor immunogenicity and thus resulting in superior patient response to PD-1/PD-L1 inhibitors in NSCLC^51^. Another DeePaN unique finding is the enrichment of mutated NKX2-1 gene in the IO-beneficial subgroup. NKX2-1 is a proto-oncogene contributing to lung cancer development, literature evidences are debating the role of NKX2-1 in lung cancer prognosis, our finding supports to continue to explore its role on post-IO prognosis^60^.

There are opportunities for future work. First, in EHRs, the existence of an assay result or the design of the treatment plan for a patient can be the result of comprehensive factors including economic stabilities, educations, community and social context, et al. One aspect of future work is to include more features such as social economic conditions et al into modeling. Second, as a graph-based AI framework, DeePaN utilized both the non-linear combination of clinico-genomic features and the patient graph structure for effective subtype identification, it remains challenging to biologically interpret this process^61^. We utilized enriched clinico-genomic features derived from DeePaN-discovered patient subtypes to inform therapeutic insight, which can be improved by future work of developing more interpretable graph-AI models such as graph attention networks^62^ to understand what drives the patient stratification to inform biomarker and therapeutic insight discovery. To validate “DeePaN”-discovered patients’ subtypes to inform clinical insight, we suggest that, as many researchers have argued^61,63,64^ and the U.S. Food and Drug Administration has been advocating^65,66^ and practicing^67^, AI models should be considered as medical devices or drugs and thus the effectiveness and safety should be evaluated through randomized clinical trials, including EHR-based pragmatic trials. A future direction will be to use multi-site randomized pragmatic trials to examine the effectiveness of the identified subtypes in augmenting clinical decisions on immunotherapies. Additionally, during the translation of a model to real-world implementation, the difference between the training and the implementation cohorts may undermine model’s effectiveness and accuracy. Due to the interpretability challenges of AI models, the impact of cohort difference on model performance cannot be apriori estimated and adjusted. Instead, transfer learning and other approaches are used^68–70^. Our model has great potential in transfer learning, benefited from the highly representative training cohort. The Flatiron cancer clinico-genomics data were collected from over 270 different cancer clinics across the nation, allowing our model to capture the common relations between biomarkers and IO responses shared by these clinics. Therefore, our model has beneficial generalizability and transfer learning potential when implemented for a specific healthcare provider. There is also room for improving the GCN model to address the over-smoothing issue, i.e., indistinguishable representations of nodes in different classes^71^ by exploring latest methods^72,73^.

Future work also includes exploring how the identified subtypes can be utilized in reality. First, the clinico-genomic features enriched in the IO-beneficial vs. non-beneficial subtypes can assist clinicians to decide what clinico-genomic tests to order to inform if IO therapy shall be prescribed for a new patient. Many of these enriched clinico-genomic features are relatively easy to measure from blood lab tests or genomic tests (see Supplementary Table 2). For instance, if a patient’s genomic test shows the presence of KRAS mutation, immunotherapy might be considered as a preferred therapy based on the insight discovery from our study. Second, a new patient can be assigned to a subtype according to modeling of clinico-genomic features. Many well-established approaches can be used for such purposes. For example, the trained DeePaN model, together with the training data, can be directly used as a transductive model to predict the subtype of a new patient through transfer learning. Our robustness test results (Supplementary Note 5) suggest that the subtyping results of the DeePaN model remain stable when the cohort varies slightly. Other approaches such as label transfer^57,59^ or supervised learning can also be used to assign new patients to DeePaN-discovered IO beneficial or non-beneficial group to inform clinical decision making. Last, in our future work, we can also explore predictive modeling to directly predict a new patient’s clinical outcome, which can be synergized with the patient-subgroup findings from DeePaN. For instance, we can include the enriched features characterizing IO-beneficial vs. non-beneficial subgroups as pre-selected input features to enhance predictive modeling (see Supplementary Note 9).

Our work thus provides evidence that integrative modeling using genomics and EHR data in a graph AI framework has clinical utility in precision oncology. As a case study, we show that as an emerging IO biomarker, although TMB-high vs. TMB-non-high groups are associated better and worse post-IO outcomes respectively, the TMB-non-high group may contain a heterogeneous patient population with distinct post-IO outcomes (Fig. 4a, b). Importantly, patient subgrouping discovered from our DeePaN framework can effectively stratify the heterogeneous TMB-non-high group to identify patient subtypes with non-high TMB but beneficial IO outcomes. This highlights the potential clinical utility of our framework on augmentation of the TMB IO biomarker. Characterization of the IO beneficial vs. non-beneficial subgroups discovered by DeePaN indicates potential to inform therapeutic insight to stratify NSCLC patients on IO outcomes. The “DeePaN” approach can be potentially applied in a wide range of clinical applications. For example, by incorporating other types of treatment regimens such as targeted therapies, chemotherapies, radiotherapies et al, this methodology can be used for recommending therapies for NSCLC patients. Similarly, this approach can be applied in other cancer types or non-cancer diseases to inform precision medicine. Besides unsupervised subtyping, representation of the original clinico-genomic data in latent space from a graph embedding can also be used for supervised learning to predict disease diagnosis or prognosis, for health trajectory projection, and so on. Our approach thus paves ways in effectively using clinico-genomic graph AI modeling for diverse applications in precision medicine.

In summary, our work serves as a proof-of-concept study to demonstrate that a patient-graph-based AI framework such as GCN is feasible and effective to integrate EHR and genomic data to inform precision oncology. With the continuous advancement of various graph-building tools and graph AI methods, we will expand our work to incorporate them to continue to inform more precision-medicine questions in the future.

## Methods

### Study design

The aim of this study is to explore the feasibility and effectiveness of a data-driven, graph AI-based unsupervised framework to strategy IO-treated NSCLC patients into subgroups with distinct immunotherapy outcomes by integrating rich genomics and EHR data. To define immunotherapy outcomes, we focused on the OS of the NSCLC population since the start date of the first IO treatment. The clinical and genomic features were defined as baseline features measured before the start of the IO therapies.

This is a secondary analysis of pre-existing, de-identified, retrospective electronic medical record data and therefore IRB review is not required.

### Patient cohort and endpoint

The NSCLC IO study cohort and dataset were established from the Flatiron Health longitudinal EHR-derived database including RWE genomics and clinical data curated from the EHR data of over 270 cancer clinics representing more than 2 million active patients across the United States. The Foundation Medicine genomic testing data in this database was from January 2010 to October 2018. The inclusion criteria of the cohort were (see Supplementary Fig. 1A and Supplementary Note 1): NSCLC patients identified with International Classification of Diseases (ICD) code for lung cancer (ICD-9: 162.x; ICD-10: C34.x or C39.9)^38^, evidence of administration of checkpoint inhibitors anti-PD-1/PD-L1 agents either as monotherapy or as part of a combination regimen^38^, and with the Foundation Medicine genomic testing data available.

The endpoint is defined as the OS of post-IO treatment. The OS time was defined as the length of time from the first use of IO therapies to the event of deceased patients, or to the last follow-up date^38^.

### Clinical features and genomic features

The clinical and genomic features were defined as baseline features measured within 6 months before the start of the IO therapies. Clinical and genomic features were screened according to prior knowledge and data availability. Totally 52 clinical features and 48 genomics features were used in our work.

Clinical features included: (1) demographics: race, gender; (2) behavioral: smoking status; (3) vitals: body weight, body height, oxygen saturation in arterial blood by pulse oximetry; (4) medical history: lines of IO therapy; (5) pathological features: Eastern Cooperative Oncology Group (ECOG) performance status, cancer stage; (6) pathological staining of biomarkers: ALK, BRAF, EGFR, KRAS, ROS1, PDL1 in tumor cells, and PDL1 in tumor infiltrated lymphocytes (TIL); (7) laboratory measurements available in more than 800 patients: leukocytes, hemoglobin, platelets, hematocrit, erythrocytes, serum creatinine, urea nitrogen, alanine aminotransferase, serum sodium, serum potassium, aspartate aminotransferase, alkaline phosphatase, serum albumin, bilirubin, serum protein, lymphocytes per 100 leukocytes, calcium, lymphocytes, monocytes per 100 leukocytes, serum glucose, serum chloride, monocytes, neutrophils, basophils per 100 leukocytes, glomerular filtration rate, basophils, eosinophils per 100 leukocytes, eosinophils, serum magnesium, granulocytes per 100 leukocytes, neutrophils, lactate dehydrogenase, and serum ferritin (see Supplementary Fig. 1B for the visualization of clinical features); (8) Foundation Medicine derived features: PDL1 expression levels in tumor cells, PDL1 expression levels in TIL, TMB^38^ (high if TMB ≥20 mutations/MB; non-high if TMB <20 mutations/MB)^38^, and microsatellite instability (MSI).

Genomic features are based on tumor sequencing of FoundationOne platform, which includes full exonic coverage of 395 genes and intronic analysis for rearrangements at a depth of 500–1000×^38^. Genomic features include known and likely genomic alterations occurring in at least 50 patients at the gene level, including the following genes (sorted by frequency, see Supplementary Fig. 1C): “TP53”, “KRAS”, “CDKN2A”, “STK11”, “CDKN2B”, “EGFR”, “PIK3CA”, “LRP1B”, “MYC”, “KEAP1”, “NF1”, “NKX2-1”, “PTEN”, “SMARCA4”, “ARID1A”, “RBM10”, “RB1”, “SOX2”, “NFKBIA”, “CCND1”, “FGF3”, “FGF4”, “FGF19”, “BRAF”, “MLL2”, “ATM”, “MDM2”, “ERBB2”, “TERC”, “MET”, “SPTA1”, “FGFR1”, “RICTOR”, “MCL1”, “DNMT3A”, “ARID2”, “PRKCI”, “FAT1”, “ZNF703”, “TERT”, “APC”, “NFE2L2”, “FGF12”, “MYST3”, “FRS2”, “TET2”, “PTPRD”, and “CCNE1”.

EHRs typically have missing data. To mitigate bias, avoid artifacts, and leverage the non-linear nature of AI models, missing values in raw data were treated as new categorical levels. Briefly, all features in raw data were converted to categorical variables, with both missing and non-missing values in original data summarized into categorical levels (see later section “Additional descriptions of methods”).

### Problem formulation

Given the NSCLC patient data with clinico-genomic features, we formulate the task of patient subgrouping as a graph clustering problem on an undirected graph encoding patient–patient relationships. Specifically, patients are represented as nodes in the graph, and patients with similar clinico-genomic features are linked by edges.

It is beneficial to formulate the patient–patient relationship into a graph since both the node content (patient clinical and genomic features) and node relationships (patient–patient connectivity based on feature similarity) will be used and integrated. We model the original clinico-genomic data as a graph *G* = (*V*, *E*, *X*) with each node *v*_*i*_ ∈ *V*, $i = 1, \cdots ,n$ represents a patient, each edge *e*_*i*,*j*_ ∈ *E* represents that the corresponding two nodes *v*_*i*_,*v*_*j*_ ∈ *V* (i.e., patients) are similar, and ***x***_*i*_ ∈ *X* represents the attribute vector associated with node *v*_*i*_. The attribute vector of each node is composed of *d* clinico-genomic features of the corresponding patient such as race, gender, LDH lactate dehydrogenase measurement, mutation status of a gene, etc. Details of categorical representation of patients’ original clinico-genomic features as well as the generation of the patient similarity graph are described in later section “Additional descriptions of methods”.

Formally, the graph can be represented by two types of information, the patient content information *X* ∈ *R*^*n*×*d*^ and the graph *G* represented by its adjacent matrix *A* ∈ *R*^*n*×*n*^. Given a patient–patient graph *G*, the goal of patient subtyping is to partition the patients (i.e., nodes) into *k* disjoint subgroups {*S*_1_, *S*_2_,…,*S*_*k*_} so that patients belonging to the same subgroup are close to each other on the graph *G*, and to discover patient subgroups with differential OS outcomes after IO treatment.

### Implementation

To achieve the above-mentioned goal, we need to solve two main tasks: (1) to learn informative patient feature representation for the downstream graph clustering method to work properly; (2) to discover new patient clusters (subgroups) on the graph that have beneficial and non-beneficial outcomes after IO treatments.

#### Learn patient deep feature graph representation

To fully extract and have deep feature representation, we apply the MGAE method^39^ to exploit the patient network information. The MGAE is based on GCN^36^ and to learn the convolution feature representation on the graph structure with the node content in the spectral domain. The reason why we use MGAE as a representative method within the GCN methodology is because of the following. First, MGAE can exploit the interplay between node content and graph structure information by using a marginalization process, which is to encode content features of the graph into the deep learning framework^74^. Second, MGAE demonstrated superior performance in comparison with the variational graph autoencoder (VGAE) and multiple typical graph-based clustering methods, based on common benchmark datasets^74^. In particular, the reconstructed feature representation can be achieved by training an MGAE^39^ on this patient network using the objective function *L* as “Eq. (1)”:

1 $$L = \frac{1}{m}\mathop {\sum}\limits_{i = 1}^m {\left\| {|X - \tilde D^{ - \frac{1}{2}}\tilde A\tilde D^{ - \frac{1}{2}}\tilde X_iW} \right\|^2} + \lambda ||W||_F^2$$

where $\tilde X = \left[ {\tilde X_1, \ldots ,\tilde X_m} \right]$ represents *m* corrupted copies of the original input $X = \left\{ {{\boldsymbol{x}}_1, \ldots ,{\boldsymbol{x}}_n} \right\} \in R^{n \times d}$, $\tilde A = A + I$ is the adjacent matrix modified with self-connections, *I* is the identity matrix, $\tilde D$ is the degree matrix of $\tilde A$, *W* is trainable weights, $\left\| \cdot \right\|_F^2$ is Frobenius norm, and *λ* is the regularization coefficient.

To learn a deep feature representation of patients’ network, we built up the network in a deep layer fashion by stacking multiple layers of AEs (Fig. 1). The patients’ representation from the output (*l* − 1)-th layer *Z*^(*l*−1)^ can be then used as input of the *l*-th layer. We used the reconstructed output from the last layer as the high-level patients’ representation for downstream analysis, i.e. detection of new patient subgroups. The implementation of MGAE is based on the open-source code available at https://github.com/FakeTibbers/MGAE.

We also explored different numbers of hidden layers as a major hyper parameter tuning, including one, three, five, and ten hidden layers. We selected three hidden layers, which is able to identify more patients with significant IO beneficial and IO non-beneficial outcomes or with stronger statistical confidence than hidden layers of one, five, and ten (see Supplementary Fig. 3 and Supplementary Note 3).

Regarding other design details of our graph neural network, based on the recommended hyperparameters in similar models published by others^74^, we set the noise corruption level to be 0.4, network regularization lambda to be 1e−5, and set the number of feature maps for each hidden layer to be 275.

#### Discovery of patient subgroups

The learned representation *Z*_0_ for the patients’ graph, which is reconstructed from MGAE’s representation (integration of both content and structure information), can then be used to discover patient subgroups. We applied the spectral clustering algorithm^39^ to discover patient subgroups. Before directly applying spectral clustering, we refine the reconstructed representation *Z*_0_ as follows:

i. Apply a linear kernel function to achieve *Z*_1_ as described by “Eq. (2)” to learn the pairwise relationship for the patient node;
  2 $$Z_1 = Z_0Z_0^T$$
ii. Ensure the representation is symmetric and nonnegative, and we achieved normalized Laplacian *Z*_2_ as described by “Eq. (3)”

3 $$Z_2 = \frac{1}{2}\left( {\left| {Z_1} \right| + \left| {Z_1^T} \right|} \right)$$

New clusters (i.e. patient subgroups) were then identified using a spectral clustering algorithm, which was done by running k-means on the top number of clusters eigenvectors of the normalized Laplacian *Z*_2_. Those clusters are identified as new patient subgroups. Spectral clustering is commonly used to perform dimensionality reduction from all the nodes in a graph and identify clusters of nodes^75^. It is probably a more natural fit to graph neural networks, which also incorporate the global information of a graph, than a “bottom-up” approach like hierarchical agglomerative clustering.

We explored different numbers of clusters (patient subgroups) as a major hyper parameter tuning, including three, five, ten clusters (Fig. 3a, Supplementary Note 2, and Supplementary Fig. 2). As demonstrated in Fig. 3a, in the setting of incorporating both genomic and clinical features, we obtained optimized results when the targeted number of clusters was set to five, which is able to identify more patients with significant IO beneficial and non-beneficial outcomes and with stronger statistical confidence.

We used the Kaplan–Meier (KM) estimate^76^ to assess if discovered subgroups have differentiable post-IO survival outcomes to inform patient stratification benefiting from IO therapies. For crossed over survival curves, log-rank test is not appropriate to calculate test statistics. Therefore, we have used Fleming-Harrington test to calculate the *P*-value for the crossed KM plots^77^ using “surv_pvalue” function from R package “survminer v0.4.7”.

#### Performance evaluation on patient subgrouping by IO outcomes

Our goal is to provide actionable insight to support the clinical decision for immune therapy, i.e. to cluster patients into subgroups and decide which subgroups are IO-beneficial or IO non-beneficial. We therefore used three measures impacting relevance to IO outcomes to assess the performance by a volcano plot (see Fig. 2c as an example). These criteria were (1) difference of median survival times between an identified cluster and the overall cohort as the baseline, with positive values corresponding to the tendency of IO beneficial outcomes and negative values corresponding to the tendency of IO non-beneficial outcomes (*x* axis); (2) statistical significance of the observed survival difference between an identified cluster and the overall cohort as the baseline (*y* axis); and (3) percentage of patients clearly assigned to significant IO beneficial and IO non-beneficial clusters using a FDR cutoff of 0.05.

A better performance corresponds to identify more patients with significant IO beneficial and non-beneficial outcomes, with stronger statistical significance, and with bigger median survival difference in comparison with the overall cohort as the baseline.

### Additional descriptions of methods

#### Clinico-genomic feature encoding and defining linked patients

In the DeePaN modeling, patients are represented as nodes in the graph with associated clinico-genomic features, and patients with similar clinico-genomic features are linked by edges. The node features are encoded by categorical feature vectors *X*. In particular, the genomic features are binary encoding, i.e. if a patient carries one or more known or likely genetic alternations in a gene, the corresponding gene feature is 1; otherwise, 0. For numerical features, we used the high- and low-bound measurement annotations provided by EHRs to bin the numerical features into categorical features. For example, a patient has the hemoglobin measurement as 8.3 g per deciliter, the low- and high-bound references for hemoglobin are 14 and 18 g per deciliter, respectively. Since it falls between two bounds, it is categorized as the “normal” class. There are 100 clinico-genomic features included, which are encoded as 275 feature dimensions. The two nodes are connected if the node feature vectors are similar. Here we employed cosine similarity to define similarity^35^. The reasons to use cosine similarity is as below. First, cosine similarity has been successfully used to estimate patient similarity based on EHRs^35^. Second, the usage of cosine similarity for binary attributes is supported by multiple literature recommendations^78–80^. We then used the cosine similarity of 0.5 as an empirical cutoff. If cosine similarity is less than 0.5, then there is not a link between two nodes; otherwise, connected. The similarity threshold 0.5 is chosen based on previous literatures’ recommendation^28,35^.

#### Missing data handling

EHRs typically have missing data. To mitigate bias, avoid artifacts, and leverage the non-linear nature of AI models, missing values in raw data were treated as new categorical levels. Briefly, all features in raw data were converted to categorical variables, with both missing and non-missing values in original data summarized into categorical levels. This approach, comparing with imputation, provides many advantages. (1) Better use of the RWE data. EHR data are often informatively censored, with data availability patterns associated with patients’ health status, access to healthcare, and clinical decisions. Our approach allowed such valuable information being intuitively captured, represented, and utilized in our model. (2) Mitigates artifacts and biases. Imputation approaches rely on information from non-missing values from closely associated features. These features often show similar data missing patterns in RWE data, known as structural data missing. For example, all lab results generated from the basic metabolic panel have the same availability pattern. This unique challenge undermines the efficiency of data imputation and exaggerates artifacts^81^. (3) Suitable for AI models. One major concern of categorizing missing values instead of imputing them is the bias and artifacts in linear models. The intrinsic nonlinear nature of AI models allows effective leveraging such data representation. Therefore, in our work, we categorize missing values to better reserve useful information and avoid artifacts and biases.

### Reporting summary

Further information on research design is available in the Nature Research Reporting Summary linked to this article.

## Supplementary information

Supplementary Information

Reporting Summary

## Data Availability

The data used in this manuscript were obtained from a de-identified clinico-genomic EHR Database generated and maintained by Flatiron Health (Flatiron Health Inc, New York, NY). Flatiron Health is subject to the requirements of the Health Insurance Portability and Accountability Act of 1996 (HIPAA) including appropriate de-identification of patients. The authors do not have permission to give public access to the study dataset. Please refer any questions or requests regarding data used in this manuscript to the following email address: published-research-data-requests@flatiron.com.

## Code availability

All tools and statistical methods used in our analysis have been described in “Methods” including access of open-source code. Additional statistical codes and machine learning implementations will be made available upon request submitted to the corresponding authors.

## References

[1] Lee CK (2017). Checkpoint inhibitors in metastatic EGFR-mutated non–small cell lung cancer—a meta-analysis. J. Thorac. Oncol..

[2] Aguiar P (2017). The effect of PD-L1 testing on the cost-effectiveness and economic impact of immune checkpoint inhibitors for the second-line treatment of NSCLC. Ann. Oncol..

[3] Langer CJ (2015). Emerging immunotherapies in the treatment of non–small cell lung cancer (NSCLC): the role of immune checkpoint inhibitors. Am. J. Clin. Oncol..

[4] James CD (1988). Clonal genomic alterations in glioma malignancy stages. Cancer Res..

[5] Simon R, Geyer S, Subramanian J, Roychowdhury S (2016). The Bayesian basket design for genomic variant-driven phase II trials. Semin Oncol..

[6] Habashy HO (2010). Transferrin receptor (CD71) is a marker of poor prognosis in breast cancer and can predict response to tamoxifen. Breast Cancer Res. Treat..

[7] Miller VA (2004). Bronchioloalveolar pathologic subtype and smoking history predict sensitivity to gefitinib in advanced non-small-cell lung cancer. J. Clin. Oncol..

[8] Shim HS, Lee DH, Park EJ, Kim SH (2011). Histopathologic characteristics of lung adenocarcinomas with epidermal growth factor receptor mutations in the International Association for the Study of Lung Cancer/American Thoracic Society/European Respiratory Society lung adenocarcinoma classification. Arch. Pathol. Lab. Med..

[9] Beaulieu-Jones BK, Greene CS (2016). Semi-supervised learning of the electronic health record for phenotype stratification. J. Biomed. Inform..

[10] Shinagare AB (2012). Unsuspected pulmonary embolism in lung cancer patients: comparison of clinical characteristics and outcome with suspected pulmonary embolism. Lung Cancer.

[11] Bepler G, Neumann K, Holle R, Havemann K, Kalbfleisch H (1989). Clinical relevance of histologic subtyping in small cell lung cancer. Cancer.

[12] Dai X (2015). Breast cancer intrinsic subtype classification, clinical use and future trends. Am. J. Cancer Res..

[13] Pikor LA, Ramnarine VR, Lam S, Lam WL (2013). Genetic alterations defining NSCLC subtypes and their therapeutic implications. Lung Cancer.

[14] Thomas A, Liu SV, Subramaniam DS, Giaccone G (2015). Refining the treatment of NSCLC according to histological and molecular subtypes. Nat. Rev. Clin. Oncol..

[15] Wislez M (2010). Non-mucinous and mucinous subtypes of adenocarcinoma with bronchioloalveolar carcinoma features differ by biomarker expression and in the response to gefitinib. Lung Cancer.

[16] Kim HS, Mendiratta S, Kim J, Pecot CV, Larsen JE (2013). Systematic identification of molecular subtype-selective vulnerabilities in non-small-cell lung cancer. Cell.

[17] Timms KM (2014). Association of BRCA1/2 defects with genomic scores predictive of DNA damage repair deficiency among breast cancer subtypes. Breast Cancer Res..

[18] Bergamaschi A (2006). Distinct patterns of DNA copy number alteration are associated with different clinicopathological features and gene‐expression subtypes of breast cancer. Genes Chromosomes Cancer.

[19] Spigel DR (2017). Results From the Phase III Randomized Trial of Onartuzumab Plus Erlotinib Versus Erlotinib in Previously Treated Stage III B or IV Non-Small-Cell Lung Cancer: METLung. J Clin Oncol.

[20] Shien K, Papadimitrakopoulou VA, Wistuba II (2016). Predictive biomarkers of response to PD-1/PD-L1 immune checkpoint inhibitors in non–small cell lung cancer. Lung Cancer.

[21] Sacher AG, Gandhi L (2016). Biomarkers for the clinical use of PD-1/PD-L1 inhibitors in non–small-cell lung cancer: a review. JAMA Oncol..

[22] Chang WY, Knochenhauer ES, Bartolucci AA, Azziz R (2005). Phenotypic spectrum of polycystic ovary syndrome: clinical and biochemical characterization of the three major clinical subgroups. Fertil. Steril..

[23] Weatherall M (2009). Distinct clinical phenotypes of airways disease defined by cluster analysis. Eur. Respir. J..

[24] Shah M (2013). The clinical phenotypes of the juvenile idiopathic inflammatory myopathies. Medicine.

[25] Gao F (2019). DeepCC: a novel deep learning-based framework for cancer molecular subtype classification. Oncogenesis.

[26] Chen R, Yang L, Goodison S, Sun Y (2019). Deep learning approach to identifying breast cancer subtypes using high-dimensional genomic data. Bioinformatics.

[27] Ronen J, Hayat S, Akalin A (2019). Evaluation of colorectal cancer subtypes and cell lines using deep learning. Life Sci. Alliance.

[28] Miotto R, Li L, Kidd BA, Dudley JT (2016). Deep patient: an unsupervised representation to predict the future of patients from the electronic health records. Sci. Rep..

[29] Rajkomar A (2018). Scalable and accurate deep learning with electronic health records. NPJ Digit. Med..

[30] Fogel AL, Kvedar JC (2018). Artificial intelligence powers digital medicine. NPJ Digit. Med..

[31] Madani A, Arnaout R, Mofrad M, Arnaout R (2018). Fast and accurate view classification of echocardiograms using deep learning. NPJ Digit. Med..

[32] Katsuki, T. et al. Feature extraction from electronic health records of diabetic nephropathy patients with convolutioinal autoencoder. In *The Workshops of the Thirty-Second AAAI Conference on Artificial Intelligence*, (ed. McIlraith S.), (AAAI, 2018).

[33] Jaques, N., Taylor, S., Sano, A. & Picard, R. In *2017 Seventh International Conference on Affective Computing and Intelligent Interaction (ACII)* 202–208 (IEEE, 2017). 10.1109/ACII.2015.7344575 PMC5431070 28515966

[34] Pai S, Bader GD (2018). Patient similarity networks for precision medicine. J. Mol. Biol..

[35] Li L (2015). Identification of type 2 diabetes subgroups through topological analysis of patient similarity. Sci. Transl. Med..

[36] Kipf, T. N. & Welling, M. Semi-supervised classification with graph convolutional networks. In *The International Conference on Learning Representations* (eds. Bengio, Y. & LeCun, Y.) (ICLR, 2017).

[37] Zhou, J. et al. Graph neural networks: a review of methods and applications. Preprint at https://arxiv.org/abs/1812.08434 (2018).

[38] Singal G (2019). Association of patient characteristics and tumor genomics with clinical outcomes among patients with non–small cell lung cancer using a clinicogenomic database. JAMA.

[39] Wang, C., Pan, S., Long, G., Zhu, X. & Jiang, J. In *Proceedings of the 2017 ACM on Conference on Information and Knowledge Management* 889–898 (ACM, 2017).

[40] Maaten Lvd, Hinton G (2008). Visualizing data using t-SNE. J. Mach. Learn. Res..

[41] McInnes, L., Healy, J. & Melville, J. Umap: uniform manifold approximation and projection for dimension reduction. Preprint at https://arxiv.org/abs/1802.03426 (2018).

[42] Hubert L, Arabie P (1985). Comparing partitions. J. Classification.

[43] Allgäuer M (2018). Implementing tumor mutational burden (TMB) analysis in routine diagnostics—a primer for molecular pathologists and clinicians. Transl. Lung Cancer Res..

[44] Goodman AM (2017). Tumor mutational burden as an independent predictor of response to immunotherapy in diverse cancers. Mol. Cancer Ther..

[45] U.S. Food and Drug Administration. FDA approves atezolizumab for first-line treatment of metastatic NSCLC with high PD-L1 expression. http://www.fda.gov/drugs/resources-information-approved-drugs/fda-approves-atezolizumab-first-line-treatment-metastatic-nsclc-high-pd-l1-expression (2020).

[46] U.S. Food and Drug Administration. FDA expands pembrolizumab indication for first-line treatment of NSCLC (TPS ≥1%). http://www.fda.gov/drugs/fda-expands-pembrolizumab-indication-first-line-treatment-nsclc-tps-1 (2019).

[47] U.S. Food and Drug Administration. FDA approves nivolumab plus ipilimumab for first-line mNSCLC (PD-L1 tumor expression ≥1%). http://www.fda.gov/drugs/drug-approvals-and-databases/fda-approves-nivolumab-plus-ipilimumab-first-line-mnsclc-pd-l1-tumor-expression-1 (2020).

[48] Karantanos T, Karanika S, Seth B, Gignac G (2019). The absolute lymphocyte count can predict the overall survival of patients with non-small cell lung cancer on nivolumab: a clinical study. Clin. Transl. Oncol..

[49] Kargl, J. et al. Neutrophil content predicts lymphocyte depletion and anti-PD1 treatment failure in NSCLC. *JCI Insight* **4** (2019). 10.1172/jci.insight.130850 PMC6975266 31852845

[50] Soyano AE (2018). Peripheral blood biomarkers correlate with outcomes in advanced non-small cell lung Cancer patients treated with anti-PD-1 antibodies. J. Immunother. Cancer.

[51] Jeanson A (2019). Efficacy of immune checkpoint inhibitors in KRAS-mutant non-small cell lung cancer (NSCLC). J. Thorac. Oncol..

[52] Liu C (2020). The superior efficacy of anti-PD-1/PD-L1 immunotherapy in KRAS-mutant non-small cell lung cancer that correlates with an inflammatory phenotype and increased immunogenicity. Cancer Lett..

[53] van Dijk D (2018). Recovering gene interactions from single-cell data using data diffusion. Cell.

[54] Butler A, Hoffman P, Smibert P, Papalexi E, Satija R (2018). Integrating single-cell transcriptomic data across different conditions, technologies, and species. Nat. Biotechnol..

[55] Wolf FA (2019). PAGA: graph abstraction reconciles clustering with trajectory inference through a topology preserving map of single cells. Genome Biol..

[56] Baran Y (2019). MetaCell: analysis of single-cell RNA-seq data using K-nn graph partitions. Genome Biol..

[57] Stuart T (2019). Comprehensive integration of single-cell data. Cell.

[58] Wang, J. et al. scGNN: a novel graph neural network framework for single-cell RNA-Seq analyses. Preprint at https://www.biorxiv.org/content/10.1101/2020.08.02.233569v1 (2020). 10.1038/s41467-021-22197-x PMC7994447 33767197

[59] Song, Q., Su, J. & Zhang, W. scGCN: a graph convolutional networks algorithm for knowledge transfer in single cell omics. Preprint at https://www.biorxiv.org/content/10.1101/2020.09.13.295535v1.full (2020). 10.1038/s41467-021-24172-y PMC8219725 34158507

[60] Yang L (2012). Nkx2-1: a novel tumor biomarker of lung cancer. J. Zhejiang Univ. Sci. B.

[61] Wang, F., Kaushal, R. & Khullar, D. Should health care demand interpretable artificial intelligence or accept “black box” medicine? *Ann. Intern. Med*. 10.7326/M19-2548 (2019). 10.7326/M19-2548 31842204

[62] Veličković, P. et al. Graph attention networks. *The International Conference on Learning Representations* (eds. Bengio, Y. & LeCun Y.) (ICLR, 2018).

[63] Shah P (2019). Artificial intelligence and machine learning in clinical development: a translational perspective. NPJ Digit. Med..

[64] Gottlieb, S. Transforming FDA’s approach to digital health. https://www.fda.gov/news-events/speeches-fda-officials/transforming-fdas-approach-digital-health-04262018 (2018).

[65] U.S. FDA. Software as a medical device. https://www.fda.gov/MedicalDevices/DigitalHealth/SoftwareasaMedicalDevice/ucm20086412.htm (2018).

[66] FDA. Digital Health Innovation Action Plan. https://www.fda.gov/media/106331/download (2018).

[67] U.S. FDA. FDA permits marketing of artificial intelligence-based device to detect certain diabetes-related eye problems. https://www.fda.gov/newsevents/newsroom/pressannouncements/ucm604357.htm (2018).

[68] Allen B, Agarwal S, Kalpathy-Cramer J, Dreyer K (2019). Democratizing AI. J. Am. Coll. Radiol..

[69] Yu KH, Beam AL, Kohane IS (2018). Artificial intelligence in healthcare. Nat. Biomed. Eng..

[70] Ting DSW (2018). AI for medical imaging goes deep. Nat. Med..

[71] Li, G., Muller, M., Thabet, A. & Ghanem, B. DeepGCNs: can GCNs go as deep as CNNs? In *Proc. IEEE International Conference on Computer Vision* (eds. Lee, K. M., Forsyth, D., Pollefeys, M. & Tang, X.) 9267–9276 (IEEE, 2019).

[72] Yang, C., Wang, R., Yao, S., Liu, S. & Abdelzaher, T. Revisiting “Over-smoothing” in deep GCNs. Preprint at https://arxiv.org/pdf/2003.13663.pdf (2020).

[73] Chen, D. et al. Measuring and relieving the over-smoothing problem for graph neural networks from the topological view. In *The Thirty-Fourth AAAI Conference on Artificial Intelligence*, 3438–3445 (AAAI, 2020).

[74] Wang, C., Pan, S., Long, G., Zhu, X. & Jiang, J. In *Proc. 2017 ACM on Conference on Information and Knowledge Management* (eds. Lim, E. P. & Winslett, M.) (Association for Computing Machinery, Singapore, Singapore, 2017).

[75] Bianchi, F. M., Grattarola, D. & Alippi, C. In *Proc. 37th International Conference on Machine Learning* (eds. Singh, A., Ill, H. D. & Blei, D.) (PMLR, 2020).

[76] Bland JM, Altman DG (1998). Survival probabilities (the Kaplan-Meier method). BMJ.

[77] Fleming, T. R. & Harrington, D. P. *Counting Processes and Survival Analysis* Vol. 169 (John Wiley & Sons, 2011).

[78] Romesburg, H. C. *Cluster Analysis for Researchers* (Lulu, 2004).

[79] Menche J (2015). Disease networks. Uncovering disease-disease relationships through the incomplete interactome. Science.

[80] Han, J., Pei, J. & Kamber, M. *Data Mining: Concepts and Techniques* (Elsevier, 2011).

[81] Chen H (2016). Relational network for knowledge discovery through heterogeneous biomedical and clinical features. Sci. Rep..

