# Supplementary material for: DeePaN: deep patient graph convolutional network integrating clinico-genomic evidence to stratify lung cancers for immunotherapy
Source: NPJ Digit Med. 2021 Feb 2;4:14. doi: 10.1038/s41746-021-00381-z (PMC7854753; doi:10.1038/s41746-021-00381-z)
Supplement: Supplementary file 1 — Supplementary Information [file 41746_2021_381_MOESM1_ESM.pdf]

Supplementary Information

Supplementary Note 1: Cohort characteristics

The cohort selection process, baseline demographic and pathologic characteristics is shown in Supplementary Fig. 1: **(A)** shows how the cohort was identified with inclusion and exclusion criteria. **(B)** shows the clinical features, such as hemoglobin, erythrocytes, hematocrit, etc. They can be categorized as molecular pathological features, blood test, and demographic behavioral and vital pathologic features. **(C)** shows the genomic features visualized by the waterfall plot of gene mutation profiling. Each row represents a gene and each column represents a patient. The mutation type can be SV (structural variant), CN (copy number variations), and RE (rearrangement). The potentially actionable somatic mutations found in this study are consistent with prior studies[1-3].

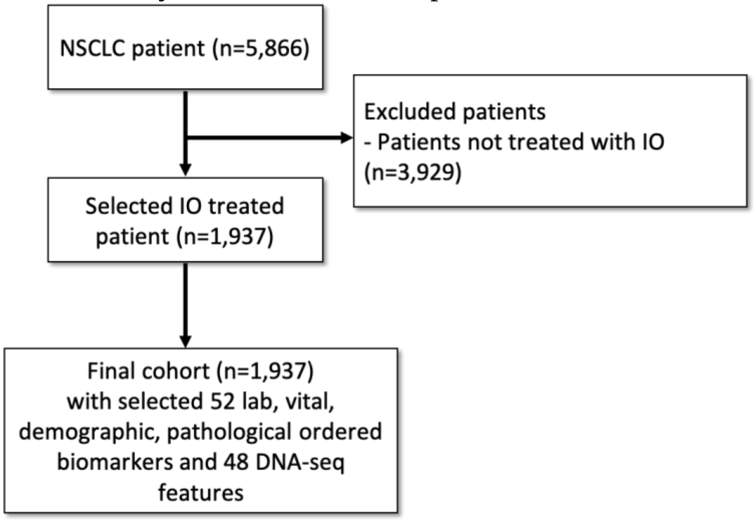

(1A)

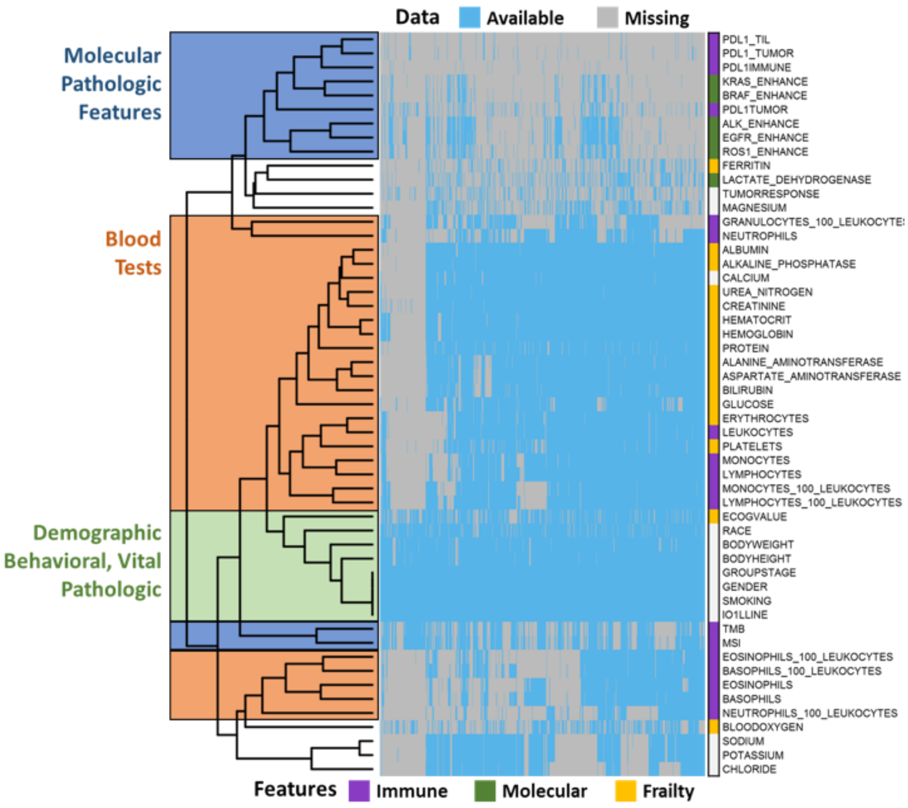

(1B)

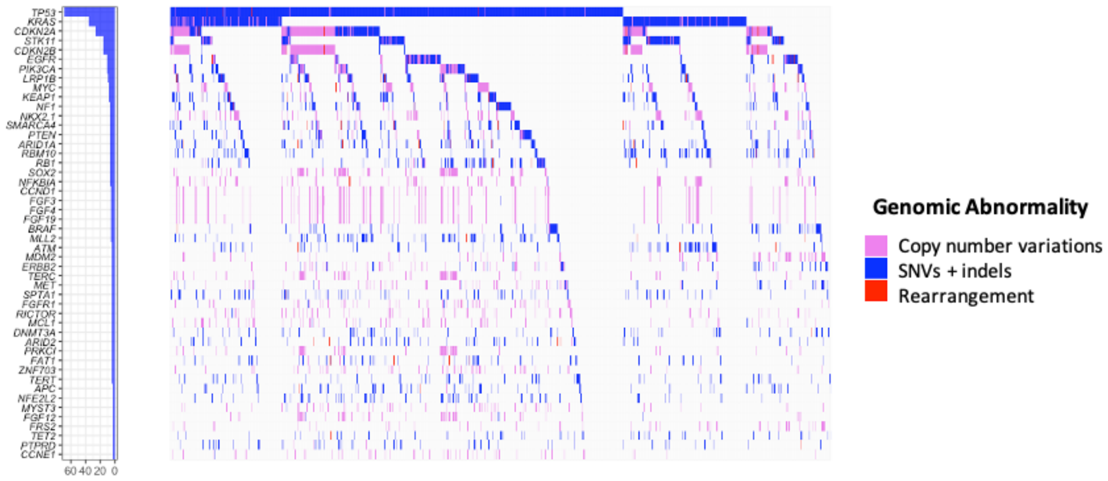

(1C)

Supplemental Fig. 1: NSCLC IO treated patient cohort and visualization of their clinical and genomic features A) Cohort Identification: an illustration of how patient cohort was identified using inclusion and exclusion criteria in this study. **B)** visualization of clinical and genomic features in the study cohort. Features are categorized into molecular pathology features, blood test features, etc. Gray color indicates missingness in the feature. Note that “Tumor response” is not included as an input feature. **C)** Waterfall plot of DNA alterations in the study cohort. The genes are sorted based on frequency.

Supplementary Note 2: Kaplan-Meier survival plots of patient subgroups at different settings.

2A: KM plots for three patient subgroups from DeePaN

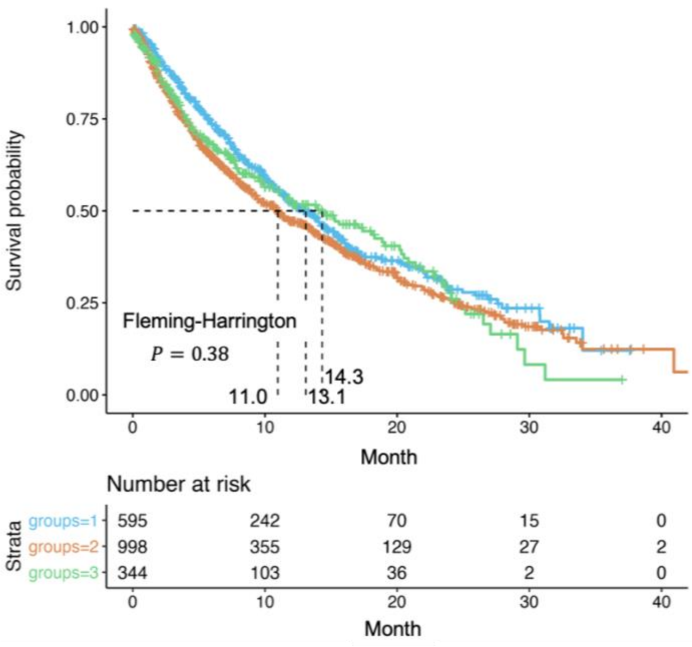

2B: KM plots for ten patient subgroups from DeePaN

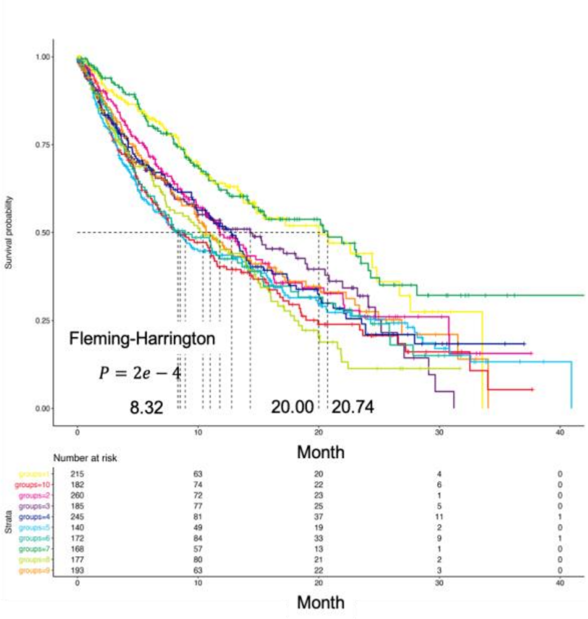

2C: KM plots for five patient subgroups from T-SNE

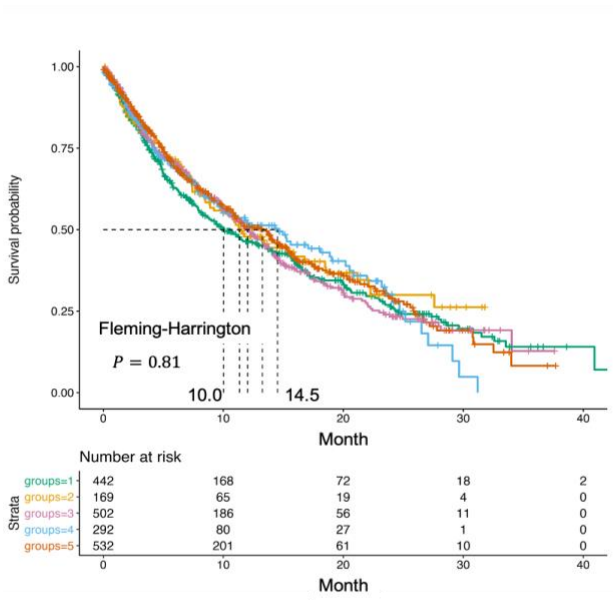

2D: KM plots for five patient subgroups from UMAP

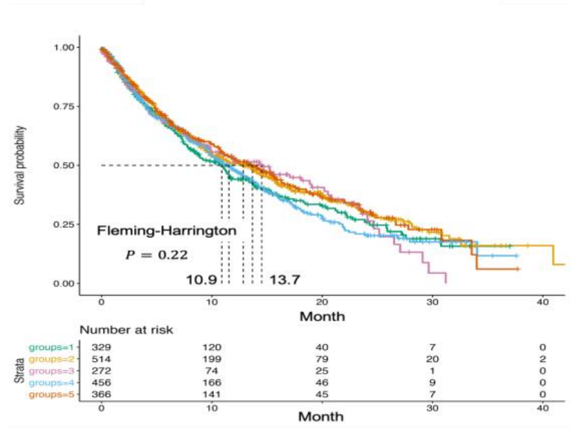

2E: KM plots for five patient subgroups from Autoencoder (AE)

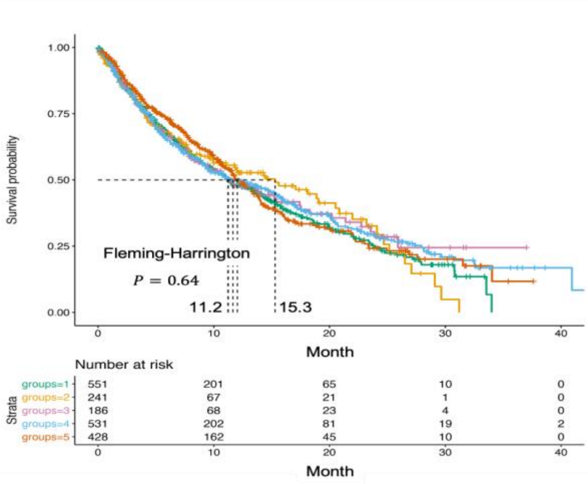

2F: KM plots for five patient subgroups from Denoising Autoencoder (DAE)

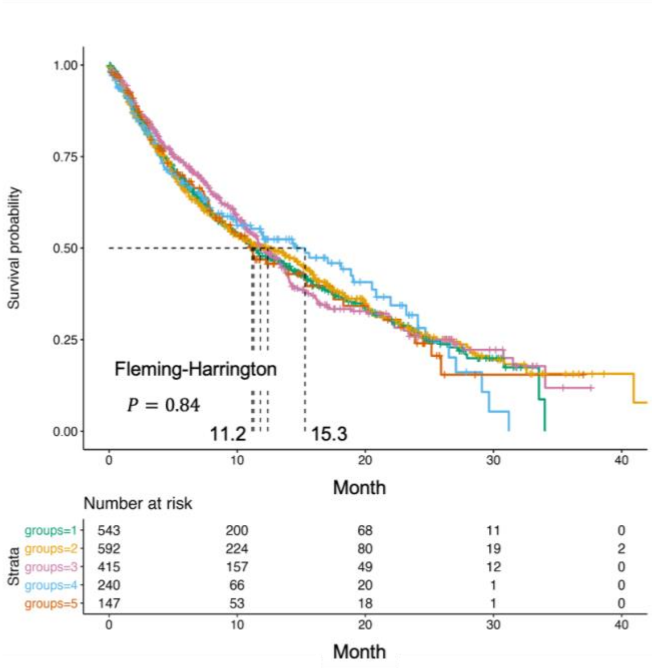

Supplementary Fig. 2: Kaplan-Meier survival plots of patient subgroups at different settings. For crossed over survival curves, log-rank test is not appropriate to calculate test statistics. Therefore we have used Fleming-Harrington test[4] calculate the p-value for the crossed Kaplan-Meier survival plots.

Supplementary Note 3: Exploration of different numbers of hidden layers for hyperparameter tuning

We explored different number of hidden layers for our GCN, including one, three, five, and ten hidden layers. As demonstrated in Supplementary Fig. 3, we obtained better results when the number of hidden layers is set to three, which is able to identifying more patients with significant IO beneficial and IO non-beneficial outcomes or with stronger statistical confidence than hidden layers of one, five, and ten.

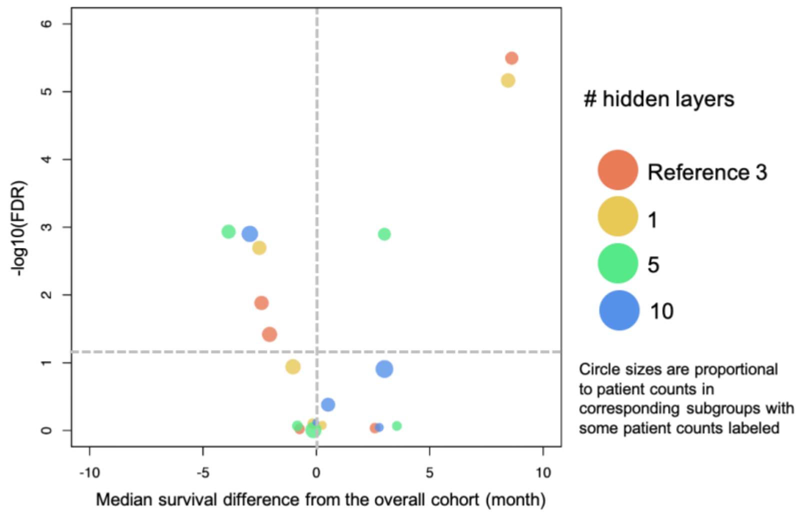

Supplementary Fig. 3: Setting hidden layer number to three for GCN has better performance than hidden layers of one, five, and ten by identifying more patients with significant IO beneficial and IO non-beneficial outcomes or with stronger statistical confidence. We explored one, three, five, and ten hidden layers with three layers as reference. We used a volcano plot for performance comparison. Each bubble represents a patient subgroup, the X axis represents the difference of the estimated median survival times between a patient subgroup and the overall cohort as control. The vertical line marked zero median survival difference, with bubbles on the right of the vertical line showing the tendency of beneficial IO outcomes and bubbles on the left showing the tendency of IO non-beneficial outcomes. Y axis is the –log10(FDR) of the corresponding log-rank test between a subgroup vs the overall cohort with multiple-comparison adjustment by Benjamini-Hochberg procedure, representing the statistical significance of the observed survival difference. The horizontal dashed line marked the statistical significance cutoff of FDR of 0.05.

Supplementary Note 4: The clinico-genomic "DeePaN" framework can identify patients with non-high TMB but with beneficial post-IO outcomes

To assess if the better post-IO survival patient subgroup discovered by DeePan (green group in Fig. 4b with non-high TMB) has clinical-relevant beneficial IO outcomes, we

used three recently FDA approved NSCLC IO trials in 2019 and 2020 as references. In these 3 recently FDA approved IO treated NSCLC trials, the Median overall survival (OS) for IO treated groups vs control groups are 17.1 months versus 14.9 (nivolumab plus ipilimumab[5]), 20 months and 12.2 months (pembrolizumab[6]), and 20.2 months and 13.1 months (atezolizumab[7]), respectively. As shown in Fig. 4b, DeePaN discovered two subgroups with median survival of 20.8 months vs 10.8 months respectively. The better survival group in Fig. 4b has the median survival of 20.8 months, which is comparable with the median survivals of the IO treated groups in these recent FDA approved IO trials and therefore demonstrated clinical-relevant IO beneficial outcomes.

Supplementary Note 5: DeePaN based patient clustering are generally robust

In this study, we would like to demonstrate the feasibility and application of GCN to patient subtyping detection. To test the robustness of our approach, we performed a ten-round "Adjusted Rand Index" test[8], which is a commonly used evaluation method for clustering robustness. For each round, we randomly removed 5% of patients. That would randomly remove 97 patients from the original dataset. Now the reduced cohort has 1,840 patients. Then, we re-ran DeePaN framework using the reduced dataset to get new five subgroups. For each new subgroup, we calculated the "Adjusted Rand Index" against the reference clustering labels (i.e. the original clustering results reported in the manuscript). The result is shown in the following, which indicates that in each round the clustering outcome matches reasonably well with the original clustering outcomes reported in the manuscript. The overall results are generally robust with the mean adjusted rand index of 0.91.

	1	2	3	4	5	6	7	8	9	10	mean
Adjusted Rand Index	0.9251	0.9254	0.9447	0.9179	0.8761	0.8754	0.8577	0.9254	0.9238	0.9219	0.9093

- Note: the Adjusted Rand Index gives a value between -1 and 1, where 0 means random labeling and 1 means perfect match between two clustering results.

Supplementary Note 6: DeePaN shows better performance than k-medoids clustering on identification of significant IO beneficial and non-beneficial subgroups

We compared DeePaN against k-medoids clustering using cosine similarity. The comparison uses performance evaluation on identification of significant IO beneficial and non-beneficial subgroups as below.

As described in the method section of the manuscript, since our goal is to provide actionable insight to support the clinical decision for immune therapy, i.e. to cluster patients into subgroups and decide which subgroups are IO beneficial vs IO non-beneficial. We therefore used three measures impacting relevance to IO outcomes to assess the performance in a volcano plot (see Supplementary Fig. 4 below as an example). These criteria were 1) difference of median survival times between an identified cluster and the overall cohort as the baseline, with positive values corresponding to the tendency of IO beneficial outcomes and negative values corresponding to the tendency of IO non-beneficial outcomes (x axis); 2) statistical significance of the observed survival difference between an identified cluster and the overall cohort as the baseline (y axis); and 3)

percentage of patients clearly assigned to significant IO beneficial and IO non-beneficial clusters using a P-value cutoff of 0.05.

A better performance corresponds to identify more patients with significant IO beneficial and non-beneficial outcomes, with stronger statistical significance, and with bigger median survival difference in comparison with the overall cohort as the baseline.

Based on the above evaluation criteria, the results showed DeePaN has better performance than k-medoids by identifying more patients with significant IO beneficial and non-beneficial outcomes and with stronger statistical confidence (Supplementary Fig. 4). In particular, the IO beneficial subgroup identified by DeePaN is more significant than k-medoids clustering (circles located in the top right corner, with P-value of 3.2×10^{-6} in DeePaN and P-value of 8.7×10^{-5} in k-medoids); DeePaN also identified more patients with significant IO beneficial and non-beneficial outcomes than k-medoids (400 vs. 376 significant IO beneficial patients in DeePaN and k-medoids, respectively; 896 in total vs 534 significant IO non-beneficial patients in DeePaN and k-medoids, respectively).

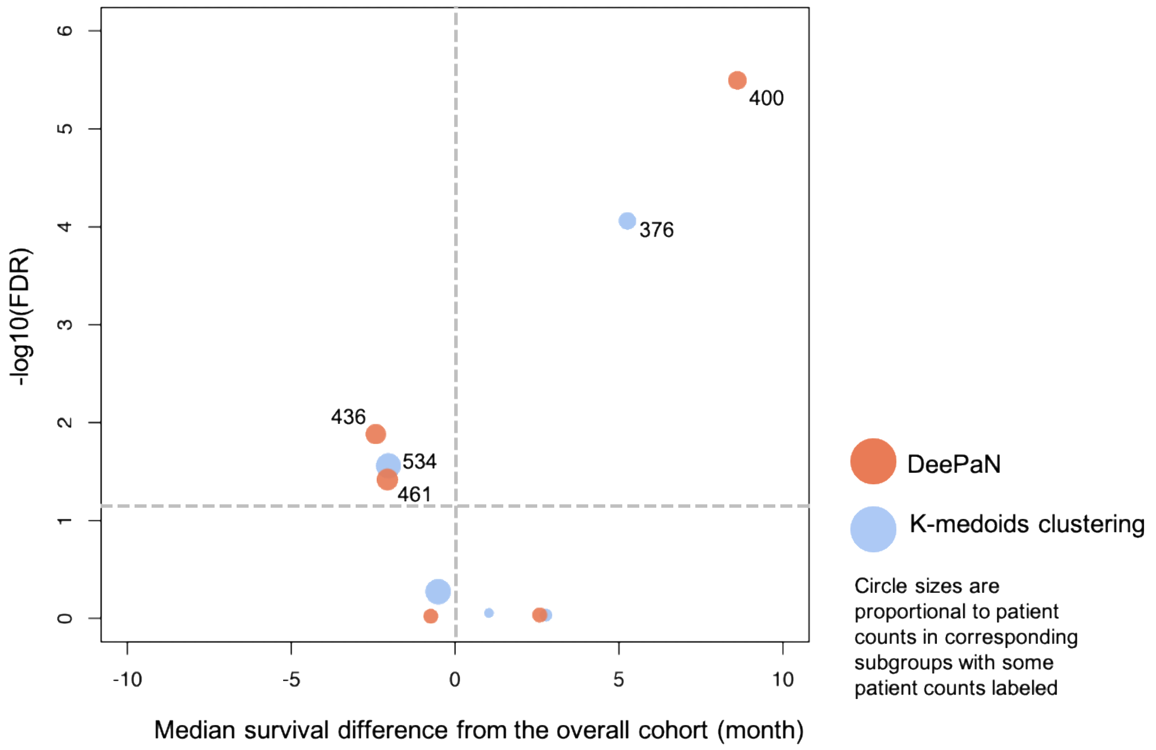

Supplementary Fig. 4: DeePaN outperforms k-medoids clustering on identification of more patients with significant IO beneficial and IO non-beneficial outcomes and with stronger statistical confidence. We used a volcano plot for performance comparison. Each bubble represents a patient subgroup, the X axis represents the difference of the estimated median survival times between a patient subgroup and the overall cohort as baseline. The vertical line marked zero median survival difference, with bubbles on the

right of the vertical line showing the tendency of beneficial IO outcomes and bubbles on the left showing the tendency of IO non-beneficial outcomes. Y axis is the –log10(FDR) of the corresponding log-rank test between a subgroup vs the overall cohort with multiple-comparison adjustment by Benjamini-Hochberg procedure, representing the statistical significance of the observed survival difference. The horizontal dashed line marked the statistical significance cutoff of FDR of 0.05.

Supplementary Note 7: Design details of our graph neural network in comparison with other design alternatives.

We use marginalized graph autoencoder (MGAE) as an implementation of our graph neural network (GCN). We compared the performances of MGAE with other related AI alternatives including the graph autoencoder (GAE), autoencoder (AE), and the denoising Autoencoder (DAE). The design details of these AI models are as below.

For marginalized graph autoencoder (MGAE), after exploring different numbers of hidden layers and different numbers of patient clusters as two major hyperparameter tunings, we selected three hidden layers and set the number of clusters to be five to optimize performance. Based on literature recommendation[9], for MGAE we set noise corruption level to be 0.4, lambda to be 1e-5 as regularization for the network training, and hidden layer node number to be 275. For GAE, it used the same settings as the MGAE, except there is no noise added toward feature inputs. For AE and DAE, the input is a matrix, of which each row represents a patient and each column represents a set of features. The categorical features are represented as one-hot encoding. The input layer contains 275 features, the embedding layer for DAE or AE contains three hidden layers, and each layer contains 100 nodes. The output layer is 275 features. For DAE, the noise (drawn from the normal distribution with zero mean and 1 standard distribution; applied 0.5 times multiplication; clipped between 0 and 1) is added to the dataset. Then the embeddings were taken and a k-mean clustering was applied to get the clustering results. To compare with MGAE, we set the number of clusters to be five for all the other methods.

The autoencoder (AE) network architecture in our experiment is shown as follows. Denoising autoencoder (DAE) network architecture is the same as AE, except for an additional component for corrupting the data.

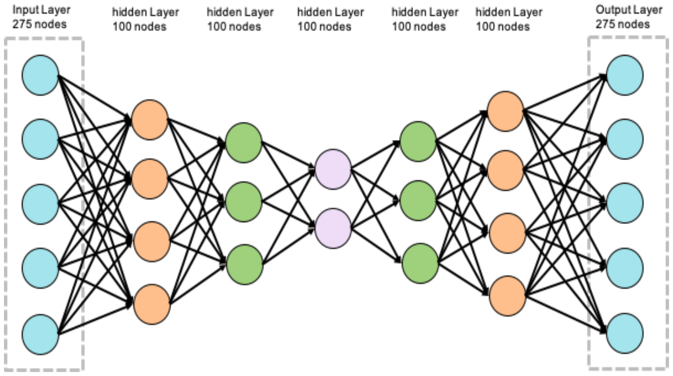

Supplementary Note 8: Explanation of the difference between Fig. 3a and Supplementary Table 2

The difference between Fig. 3a and the enrichment analysis (Supplementary Table 2) highlighted the advantage of using non-linear models such as DeePaN to address precision oncology problems in complex diseases such as NSCLC. The associations between clinico-genomic factors and patient outcome on immunotherapy are complex and sometimes highly non-linear. Enrichment analysis (Supplementary Table 2) demonstrated that, among all significant genomic features, at least three of them (KRAS, NKX2, and TMB) were independently and significantly associated with the DeePaN discovered IO beneficial vs. non-beneficial subgroups. Fig. 3a suggested that, beyond the simple and direct associations between these three genomic features and the two discovered patient subtypes, complex and non-linear combinations of genomic features with other genomic or clinical features are able to further distinguish IO beneficial and non-beneficial patients. Indeed when genomic and clinical features are directly assessed using the logrank test only TMB is deemed significantly associated to immunotherapy outcome (see Supplementary Table 3). Our DeePaN based redefinition of IO beneficial/IO non-beneficial patient subgroups focuses on grouping clinico-genomic similar patients instead of relying regression on IO outcomes. As the caveat of our current proof-of-concept GCN model, the exact feature usage and predictive rules used by our GCN method are not visible. However, as an ensemble strategy our attempt to reconstruct biomarker importance through univariate-based enrichment analysis of comparing GCN discovered patient subgroups reveals significance from two additional genomic markers, KRAS and NKX2, along with TMB. These three genetic markers alone do not explain the strong dependency GCN model on incorporating genetic data as an essential input, but it is likely additional genetic features and especially feature combinations are being used in the GCN for efficient patient subtype discovery, just each of those additional genomic features alone does not pass our univariate-based enrichment analysis significance.

Our current work is a proof-of-concept study to show graph AI methods such as GCN is feasible and effective to integrated RWE clinico-genomic evidence to inform precision oncology insight. In our future work, we plan to develop more interpretable graph-AI models such as graph attention networks to understand how these genomic and clinical features in synergy drives the patient stratification to inform biomarker and therapeutic insight discovery.

Supplementary Note 9: Discussion of patient subgrouping vs predictive modeling

There are multiple applications to use machine learning for therapeutic insight discovery using clinico-genomic data, including i) building a predictive model to directly predict a patient’s clinical or disease outcome[10] and ii) informing patient/disease heterogeneity for precision medicine by discovering patient subtypes such as subgroup modeling[10,11]. Our work falls into the scope of ii).

For i) directly building a predictive model, it typically aims to build one general model to predict patients’ clinical outcomes for the entire patient population with a focus on prediction accuracy ; for ii) patient subtype discovery, it typically aims to discover patient

subgroups as well as to characterize individual subgroups to inform precision medicine instead of characterizing the entire patient population.

Because lung cancer is a heterogenous disease, our work focuses on discovery of patient subgroups to inform precision oncology.

Actually, building a predictive model and patient subtype modeling tend to be complementary and can be combined for synergy. For instance, the enriched clinico-genomic features characterizing IO beneficial vs non-beneficial subgroups derived from DeePaN can serve as pre-selected input features to enhance predictive modeling.

Supplementary Note 10: Defining the IO beneficial vs non-beneficial subgroups discovered by DeePaN base on the clinical relevance

To assess if the IO beneficial vs non-beneficial patient subgroups (green group vs red group in Fig. 2d) discovered by DeePaN has clinical relevance, we used three recently FDA approved NSCLC IO trials in 2019 and 2020 as references. In these 3 recently FDA approved IO treated NSCLC trials, the median overall survival (OS) for IO treated groups vs control groups are 17.1 months versus 14.9 (nivolumab plus ipilimumab[5]), 20 months and 12.2 months (pembrolizumab[6]), and 20.2 months and 13.1 months (atezolizumab[7]), respectively. As shown in Fig. 2d, DeePaN discovered two subgroups with median survival of 20.35 months vs 9.42 months respectively. The better survival group in Fig. 2d has the median survival of 20.35 months, which is comparable with the median survivals of the IO treated groups in these recent FDA approved IO trials and therefore demonstrated clinical-relevant IO beneficial outcomes. Thus this better survival group is defined as the IO beneficial subgroup. The worse survival group has about 10 months less median survival in comparison with the better survival group, therefore we define them as IO non-beneficial subgroup.

Supplementary Table 1: Baseline demographic and pathologic characteristics for the overall IO cohort, IO beneficial subgroup, and IO non-beneficial subgroup.

Characteristics	All	IO Beneficial Subgroup	IO Non-beneficial Subgroups
number of patients	1,937	400	897
Age (year)			
Median, MAD	69.0, 10.4	68.0, 10.4	68.0, 10.4
Range	26.0-85.0	27.0-85.0	34.0-85.0
Sex: Number (%)			
Male	984 (50.8)	148 (37.0)	522 (58.2)
Female	953 (49.2)	252 (63.0)	375 (41.8)
Race: Number (%)			
African American	144 (7.4)	31 (7.8)	80 (8.9)
White	1,428 (73.7)	289 (72.3)	648 (72.2)
Asian	46 (2.4)	10 (2.5)	19 (2.1)
Other Race	143 (7.4)	41 (10.3)	68 (7.6)
Histology: Number (%)			
Non-squamous cell carcinoma	1,443 (74.5)	329 (82.2)	601 (67.0)
Squamous cell carcinoma	419 (21.6)	62 (15.5)	259 (28.9)
NSCLC histology NOS	75 (3.8)	9 (2.3)	37 (4.1)
Stage: Number (%)			
Stage I	164 (8.5)	45 (11.3)	69 (7.7)
Stage II	122 (6.3)	30 (7.5)	55 (6.1)
Stage III	372 (19.2)	95 (23.8)	176 (19.6)
Stage IV	1,241 (64.1)	225 (56.3)	571 (63.7)
ECOG Score: Number (%)			
0	375 (19.4)	104 (26.0)	129 (14.4)
1	856 (44.2)	176 (44.0)	430 (47.9)
2	273 (14.1)	27 (6.8)	149 (16.6)
3	50 (2.6)	6 (1.5)	27 (3.0)
4	2 (0.1)	0 (0.0)	2 (0.2)
Smoking Status: Number (%)			
History of smoking	1,657 (85.5)	342 (85.5)	775 (86.4)
No history of smoking	276 (14.2)	57 (14.3)	120 (13.4)
Previous Treatment: Number (%)			
No	718 (37.1)	170 (42.5)	228 (25.4)
Yes	1,219 (62.9)	230 (57.5)	669 (74.6)

Eastern Cooperative Oncology Group (ECOG)
MAD: Median Absolute Deviation

Supplementary Table 2: Enriched clinical and genomics characteristics differentiating the IO beneficial vs non-beneficial subgroups. Enriched features with significant difference between the IO beneficial and non-beneficial subgroups were identified by chi-square test (two-sided) with multiple-comparison adjustment of FDR less than 0.05.

Feature Name	FDR	IO Beneficial Subgroup	IO Non-beneficial Subgroup
HEMOGLOBIN	1.60E-178	Normal	Low
HEMATOCRIT	2.49E-168	Normal	Low
ERYTHROCYTES	5.12E-130	Normal	Low
ALBUMIN	1.66E-23	Normal	Low
FERRITIN	5.39E-17	Normal	High
LYMPHOCYTES	2.92E-14	Normal	Low
GENDER	2.67E-10	Female	Male
NEUTROPHILS	2.52E-08	Normal	High
BASOPHILS	4.01E-06	Abnormal	Normal
PROTEIN	6.31E-06	Normal	Abnormal
MONOCYTES	3.70E-05	Normal	High
LYMPHOCYTES %	6.99E-04	Normal	Low
PLATELETS	1.16E-03	Normal	High
GRANULOCYTES %	1.35E-03	Normal	High
LEUKOCYTES	5.54E-03	Normal	High
EOSINOPHILS	1.29E-02	Normal	Abnormal
NKX2-1	2.04E-02	Altered	Wild-type
KRAS	3.55E-02	Altered	Wild-type
CALCIUM	3.61E-02	Normal	Abnormal
GLUCOSE	4.74E-02	Normal	High
TMB	4.90E-02	High	Low

Supplementary Table 3: Comparison of DeePaN enriched clinico-genomic features with log-rank test.

Clinico-genomic Features	DeePaN enrichment (FDR)[1]	Survial significance(FDR)[2]	IO Beneficial associations	IO non-benficial associations	Literature evidence	Link to literature evidence
HEMOGLOBIN	1.60E-178	1.71E-07	Normal	Low	Pre-treatment hemoglobin levels are an independent prognostic factor in patients with non-small cell lung cancer (prognostic factor)	https://www.ncbi.nlm.nih.gov/pmc/articles/PMC5995083/
HEMATOCRIT	2.49E-168	1.12E-05	Normal	Low	Low Hematocrit Is a Strong Predictor of Poor Prognosis in Lung Cancer Patients (prognostic factor)	https://www.researchgate.net/publication/328362001
ERYTHROCYTES	5.12E-130	7.31E-07	Normal	Low	Low Hematocrit Is a Strong Predictor of Poor Prognosis in Lung Cancer Patients; Hematrit measures red blood cells (Erythocyties) (prognostic factor)	https://www.researchgate.net/publication/328362001
ALBUMIN	1.66E-23	1.36E-09	Normal	Low	Decreased albumin was associated with decreased survival in NSCLC (prognostic factor)	https://www.nature.com/articles/s41598-019-44653-x
FERRITIN	5.39E-17	8.85E-01	Normal	High	The serum ferritin concentration is a significant prognostic indicator of survival in primary lung cancer (prognostic factor)	https://www.ncbi.nlm.nih.gov/pubmed/11748482
LYMPHOCYTES	2.92E-14	1.00E+00	Normal	Low	The absolute lymphocyte count can predict the overall survival of patients with non-small cell lung cancer on nivolumab (relevant to IO mechanistic insight)	https://rd.springer.com/article/10.1007%2Fs12094-018-1908-2
GENDER	2.67E-10	1.00E+00	Female	Male	The overall survival was significantly better in women than men from a retrospective analysis of 12,509 cases in a Japanese Lung Cancer Registry study (prognostic factor)	https://www.ncbi.nlm.nih.gov/pubmed/20736855
NEUTROPHILS	2.52E-08	1.05E-04	Normal	High	Neutrophil content predicts lymphocyte depletion and anti-PD1 treatment failure in NSCLC (relevant to IO mechanistic insight)	https://insight.jci.org/articles/view/130850
BASOPHILS	4.01E-06	1.00E+00	Abnormal	Normal	Need to explore further	
PROTEIN	6.31E-06	8.75E-01	Normal	Abnormal	low protein associated with poor prognosis in NSCLC (prognostic factor)	https://onlinelibrary.wiley.com/doi/pdf/10.1002/ijc.28995
MONOCYTES	3.70E-05	1.00E+00	Normal	High	high monocyte count associated with poor post IO prognosis (relevant to IO mechanistic insight)	https://www.ncbi.nlm.nih.gov/pmc/articles/PMC6251165/

**LYMPHOCYTES_100_LEUKOCYTES**	6.99E-04	1.92E-01	Normal	Low	The absolute lymphocyte count can predict the overall survival of patients with non-small cell lung cancer on nivolumab: a clinical study (relevant to IO mechanistic insight)	https://rd.springer.com/article/10.1007%2Fs12094-018-1908-2
**PLATELETS**	1.16E-03	9.76E-02	Normal	High	The present study demonstrated that an increased platelet count can help to predict unfavorable outcome in lung cancer (prognostic factor).	http://ar.iiarjournals.org/content/33/4/1725.full
**GRANULOCYTES_100_LEUKOCYTES**	1.35E-03	1.78E-01	Normal	High	Need to explore further	
**LEUKOCYTES**	5.54E-03	4.28E-03	Normal	High	Evidence of High leukocytes may be potentially associated with poor survial in NSCLC (table 2 of the reference) (prognostic factor)	https://onlinelibrary.wiley.com/doi/pdf/10.1002/ijc.28995
**EOSINOPHILS**	8.24E-03	1.00E+00	Normal	Abnormal	Need to explore further	
**NKX2-1**	2.04E-02	1.00E+00	Altered	Wild-type	Hypothesis needs to be further explored as there are debating literature evidence for the prognostic role of Nkx2-1 in lung cancer	https://www.ncbi.nlm.nih.gov/pmc/articles/PMC3494024/
**KRAS**	3.55E-02	1.00E+00	Altered	Wild-type	PD-1/PD-L1 blockade monotherapy may be the optimal therapeutic schedule in NSCLC patients harboring KRAS mutations, with KRAS mutations correlating with an inflammatory tumor microenvironment and tumor immunogenicity and thus resulting in superior patient response to PD-1/PD-L1 inhibitors (relevant to IO mechanistic insight)	https://www.ncbi.nlm.nih.gov/pubmed/31644929
**CALCIUM**	3.61E-02	2.76E-03	Normal	Abnormal	In Cox proportional hazard model analysis, corrected hypercalcemia levels and alkaline phosphatase levels were determined to be risk factors affecting patients' survival time (hazard ratio [HR] = 6.828, P = 0.000; HR = 1.957, P = 0.026). (prognostic factor)	https://www.ncbi.nlm.nih.gov/pubmed/26612452

**GLUCOSE**	4.74E-02	1.17E-01	Normal	High	Targeting glucose metabolism to enhance immunotherapy in NSCLC is being explored. There is an ongoing clinical trial exploring combination therapy of (Nivolumab + Metformin) in NSCLC, further explorations are needed (potentially relevant to IO mechanistic insight).	https://www.ncbi.nlm.nih.gov/pmc/articles/PMC6603129/
**TMB**	4.90E-02	6.15E-04	High	low	Tumor mutational burden (TMB) as an emerging biomarker of response to immunotherapy in NSCLC (relevant to IO mechanistic insight)	https://www.ncbi.nlm.nih.gov/pubmed/30505709
**UREA_NITROGEN**	3.21E-01	1.48E-02	normal	abnormal	elevated BUN was associated with unfavorable survival in NSCLC (prognostic factor)	https://onlinelibrary.wiley.com/doi/pdf/10.1002/ijc.28995
**ALKALINE_PHOSPHATASE**	8.47E-01	4.09E-09	low	normal	elevation of alkaline phosphatase was a predictor for poor survival in lung cancer (prognostic factor)	https://www.ncbi.nlm.nih.gov/pubmed/26612452
**CHLORIDE**	1.00E+00	3.05E-05	normal	Abnormal	Need to be further explored. In India, NSCLC patients are at high risk of having hyponatremia at presentation and this is significantly associated with a worse outcome.	https://www.ncbi.nlm.nih.gov/pmc/articles/PMC3342719/
**RACE**	1.00E+00	2.37E-02	reported	not reported	Need to explore further	
**POTASSIUM**	1.00E+00	2.81E-02	normal	Abnormal	Need to explore further, limited literature evidence in NSCLC	https://www.ackdjournal.org/article/S1548-5595(13)00077-3/fulltext
**GROUPSTAGE**	1.00E+00	4.72E-02	early	late	Late stage NSCLC patients tend to have poor prognosis (prognostic factor)	https://www.healthline.com/health/lung-cancer-stages-survival-rates#survival-rates

[1]: chi-square test to identify enriched features in IO beneficial vs non-beneficial subgroups discovered by DeePaN.

[2]: logrank test with Kaplan- Meier estimator for features associated with survial.

21 significant features are identified by chi-square test based enrichment analysis on IO beneficial vs non-beneficial subgroups discovered by DeePaN (FDR cutoff of 0.05)

14 significant features are identified using logrank test (FDR cutoff of 0.05)

Green : 13 features found by DeePaN based enrichment analysis but not by logrank test

Blue: 8 significant features in common between DeePaN based enrichment analysis and logrank test

Orange: 6 significant features identified by logRank test but not by DeePaN based enrichment analysis

Reference:

1 Jordan, E. J., Kim, H. R., Arcila, M. E., Barron, D., Chakravarty, D. *et al.* Prospective comprehensive molecular characterization of lung adenocarcinomas for efficient patient matching to approved and emerging therapies. *Cancer discovery* **7**, 596-609 (2017).

2 Network, C. G. A. R. Comprehensive genomic characterization of squamous cell lung cancers. *Nature* **489**, 519 (2012).

3 Network, C. G. A. R. Comprehensive molecular profiling of lung adenocarcinoma. *Nature* **511**, 543 (2014).

4 Fleming, T. R. & Harrington, D. P. *Counting processes and survival analysis*. Vol. 169 (John Wiley & Sons, 2011).

5 U.S. Food and Drug Administration. *FDA approves nivolumab plus ipilimumab for first-line mNSCLC (PD-L1 tumor expression ≥1%)*, <http://www.fda.gov/drugs/drug-approvals-and-databases/fda-approves-nivolumab-plus-ipilimumab-first-line-mnsclc-pd-l1-tumor-expression-1> (2020).

6 U.S. Food and Drug Administration. *FDA expands pembrolizumab indication for first-line treatment of NSCLC (TPS ≥1%)*, <http://www.fda.gov/drugs/fda-expands-pembrolizumab-indication-first-line-treatment-nsclc-tps-1> (2019).

7 U.S. Food and Drug Administration. *FDA approves atezolizumab for first-line treatment of metastatic NSCLC with high PD-L1 expression*, <http://www.fda.gov/drugs/resources-information-approved-drugs/fda-approves-atezolizumab-first-line-treatment-metastatic-nsclc-high-pd-l1-expression> (2020).

8 Hubert, L. & Arabie, P. Comparing partitions. *Journal of Classification* **2**, 193-218, doi:10.1007/BF01908075 (1985).

9 Wang, C., Pan, S., Long, G., Zhu, X. & Jiang, J. in *Proceedings of the 2017 ACM on Conference on Information and Knowledge Management* 889–898 (Association for Computing Machinery, Singapore, Singapore, 2017).

10 Miotto, R., Li, L., Kidd, B. A. & Dudley, J. T. Deep Patient: An Unsupervised Representation to Predict the Future of Patients from the Electronic Health Records. *Sci Rep* **6**, 26094, doi:10.1038/srep26094 (2016).

11 Li, L., Cheng, W. Y., Glicksberg, B. S., Gottesman, O., Tamler, R. *et al.* Identification of type 2 diabetes subgroups through topological analysis of patient similarity. *Sci Transl Med* **7**, 311ra174, doi:10.1126/scitranslmed.aaa9364 (2015).
