# Supplementary material for: DeePaN: deep patient graph convolutional network integrating clinico-genomic evidence to stratify lung cancers for immunotherapy
Source: NPJ Digit Med. 2021 Feb 2;4:14. doi: 10.1038/s41746-021-00381-z (PMC7854753; doi:10.1038/s41746-021-00381-z)
Supplement: Supplementary file 2 — Reporting Summary [file 41746_2021_381_MOESM2_ESM.pdf]

nature research

Corresponding author(s): Bolan Linghu

Last updated by author(s): Dec 28, 2020

Reporting Summary

Nature Research wishes to improve the reproducibility of the work that we publish. This form provides structure for consistency and transparency in reporting. For further information on Nature Research policies, see our Editorial Policies and the Editorial Policy Checklist.

Statistics

For all statistical analyses, confirm that the following items are present in the figure legend, table legend, main text, or Methods section.

n/a	Confirmed	
☐	☒	The exact sample size (*n*) for each experimental group/condition, given as a discrete number and unit of measurement
☐	☒	A statement on whether measurements were taken from distinct samples or whether the same sample was measured repeatedly
☐	☒	The statistical test(s) used AND whether they are one- or two-sided<br>*Only common tests should be described solely by name; describe more complex techniques in the Methods section.*
☐	☒	A description of all covariates tested
☐	☒	A description of any assumptions or corrections, such as tests of normality and adjustment for multiple comparisons
☐	☒	A full description of the statistical parameters including central tendency (e.g. means) or other basic estimates (e.g. regression coefficient) AND variation (e.g. standard deviation) or associated estimates of uncertainty (e.g. confidence intervals)
☒	☐	For null hypothesis testing, the test statistic (e.g. *F*, *t*, *r*) with confidence intervals, effect sizes, degrees of freedom and *P* value noted<br>*Give P values as exact values whenever suitable.*
☒	☐	For Bayesian analysis, information on the choice of priors and Markov chain Monte Carlo settings
☒	☐	For hierarchical and complex designs, identification of the appropriate level for tests and full reporting of outcomes
☒	☐	Estimates of effect sizes (e.g. Cohen's *d*, Pearson's *r*), indicating how they were calculated

Our web collection on statistics for biologists contains articles on many of the points above.

Software and code

Policy information about availability of computer code

Data collection	The data used in this manuscript were obtained from a de-identified clinico-genomic EHR Database generated and maintained by Flatiron Health (Flatiron Health Inc, New York, NY). Flatiron Health is subject to the requirements of the Health Insurance Portability and Accountability Act of 1996 (HIPAA) including appropriate de-identification of patients. The authors do not have permission to give public access to the study dataset. Please refer any questions or requests regarding data used in this manuscript to the following email address: published-research-data-requests@flatiron.com.<br><br>The study was funded by AstraZeneca. AstraZeneca licensed the de-identified Flatiron Clinico-Genomic EHR Database from Flatiron Health (Flatiron Health Inc, New York, NY) for data access and usage, including supporting the analyses in this manuscript.<br><br>Data of 1,937 immunotherapy treated non-small cell lung cancer patient were retrieved from the Flatiron clinico-genomic database for study. (reference: https://jamanetwork.com/journals/jama/fullarticle/2730114)
Data analysis	All tools and statistical methods used in our analysis have been described in Methods including access of open-source code. Additional statistical codes and machine learning implementations will be made available upon request submitted to the corresponding authors.

For manuscripts utilizing custom algorithms or software that are central to the research but not yet described in published literature, software must be made available to editors and reviewers. We strongly encourage code deposition in a community repository (e.g. GitHub). See the Nature Research guidelines for submitting code & software for further information.

Data

Policy information about availability of data

All manuscripts must include a data availability statement. This statement should provide the following information, where applicable:

- Accession codes, unique identifiers, or web links for publicly available datasets
- A list of figures that have associated raw data
- A description of any restrictions on data availability

The data used in this manuscript were obtained from a de-identified clinico-genomic EHR Database generated and maintained by Flatiron Health (Flatiron Health Inc, New York, NY). Flatiron Health is subject to the requirements of the Health Insurance Portability and Accountability Act of 1996 (HIPAA) including appropriate de-identification of patients. The authors do not have permission to give public access to the study dataset. Please refer any questions or requests regarding data used in this manuscript to the following email address: published-research-data-requests@flatiron.com.

Field-specific reporting

Please select the one below that is the best fit for your research. If you are not sure, read the appropriate sections before making your selection.

☒ Life sciences ☐ Behavioural & social sciences ☐ Ecological, evolutionary & environmental sciences

For a reference copy of the document with all sections, see nature.com/documents/nr-reporting-summary-flat.pdf

Life sciences study design

All studies must disclose on these points even when the disclosure is negative.

Sample size	N= 1,937 immunotherapy treated non-small cell lung cancer (NSCLC) patients from the Flatiron clinico-genomic database
Data exclusions	NSCLC Patients not treated with immunotherapy were excluded.
Replication	The reproducibility of experiment findings was verified from a ten-round cross validation through resampling.
Randomization	Randomization is not relevant to our work as our work is not a case-control study.
Blinding	Blinding is not not relevant to our work as our work is not a case-control study.

Reporting for specific materials, systems and methods

We require information from authors about some types of materials, experimental systems and methods used in many studies. Here, indicate whether each material, system or method listed is relevant to your study. If you are not sure if a list item applies to your research, read the appropriate section before selecting a response.

Materials & experimental systems

n/a	Involved in the study	
☒	☐	Antibodies
☒	☐	Eukaryotic cell lines
☒	☐	Palaeontology and archaeology
☒	☐	Animals and other organisms
☒	☐	Human research participants
☐	☒	Clinical data
☒	☐	Dual use research of concern

Methods

n/a	Involved in the study	
☒	☐	ChIP-seq
☒	☐	Flow cytometry
☒	☐	MRI-based neuroimaging

Clinical data

Policy information about clinical studies

All manuscripts should comply with the ICMJE guidelines for publication of clinical research and a completed CONSORT checklist must be included with all submissions.

Clinical trial registration	This is a secondary analysis of pre-existing, de-identified, retrospective electronic medical record data, therefore IRB review and clinical trial registrations are not required.
Study protocol	This is a secondary analysis of pre-existing, de-identified, retrospective electronic medical record data, therefore IRB review and study protocols are not required.
Data collection	The study was funded by AstraZeneca. AstraZeneca licensed the de-identified Flatiron clinico-genomic EHR Database from Flatiron Health (Flatiron Health Inc, New York, NY) for data access and usage, including supporting the analyses in this manuscript.

Data of 1,937 immunotherapy treated non-small cell lung cancer patient were retrieved from the Flatiron clinico-genomic database for study. (reference: https://jamanetwork.com/journals/jama/fullarticle/2730114)

Outcomes

Outcomes are defined as patients' overall survival of post immunotherapy.
